# The temporal trajectories of habit decay in daily life: An intensive longitudinal study on four health‐risk behaviors

**DOI:** 10.1111/aphw.12612

**Published:** 2024-10-25

**Authors:** Robert Edgren, Dario Baretta, Jennifer Inauen

**Affiliations:** ^1^ Department of Health Psychology and Behavioral Medicine University of Bern Bern Switzerland

**Keywords:** habit, health‐risk behavior, intensive longitudinal methods, n‐of‐1, time series

## Abstract

Habits are cue‐behavior associations learned through repetition that are assumed to be relatively stable. Thereby, unhealthy habits can pose a health risk due to facilitating relapse. In the absence of research on habit decay in daily life, we aimed to investigate how habit decreases over time when trying to degrade a habit and whether this differs by four health‐risk behaviors (sedentary behavior, unhealthy snacking, alcohol consumption, and smoking). This 91‐day intensive longitudinal study included four parallel non‐randomized groups (one per behavior; *N* = 194). Habit strength was measured daily with the Self‐Report Behavioral Automaticity Index (11,805 observations) and modelled over time with constant, linear, quadratic, cubic, asymptotic, and logistic models. Person‐specific modelling revealed asymptotic and logistic models as the most common best‐fitting models (54% of the sample). The time for habit decay to stabilize ranged from 1 to 65 days. Multilevel modelling indicated substantial between‐person heterogeneity and suggested initial habit strength but not the decay process to vary by behavioral group. Findings suggest that habit decay when trying to degrade a habit typically follows a decelerating negative trend but that it is a highly idiosyncratic process. Recommendations include emphasizing the role of person‐specific modelling and data visualization in habit research.

## INTRODUCTION

Habits, centrally defined as cue–behavior associations learned through repetition (Fleetwood, 2021; Gardner, 2015; Gardner et al., 2022), may have a substantial negative impact on health through sustaining health‐risk behavior. Smoking, alcohol consumption, lack of physical activity, and unhealthy diet are leading causes of premature deaths that are preventable through behavioral change (Habib & Saha, 2010; Keeney, 2008; Muller et al., 2016). These behaviors can be influenced by underlying habits (Albery et al., 2015; Conroy et al., 2013; Gardner et al., 2021; Ray et al., 2020; Webb et al., 2009), whereby encountering the cue automatically triggers an impulse towards habitual behavior (Gardner, 2015). Previously formed habits can hinder achieving counter‐habitual behavioral change, as evidenced for example by unintentional behavioral slips (Orbell & Verplanken, 2010). Thus, weakening (or “breaking”) habits in addition to changing behavior may be necessary. However, habit decay may be challenging due to the nature of habit, in particular its key feature, automaticity. Automaticity entails cue contingency, goal independence, functionality without conscious awareness, and processing characterized by efficiency and speed (Mazar & Wood, 2018; Moors & De Houwer, 2006). This may lead habitual responses to be favored under conditions in which self‐control is depleted, such as when stressed (Neal et al., 2013; Wood & Rünger, 2016). Furthermore, factors related to the behavior itself may add complexity to the decay process. Particularly, the reward value of an unhealthy habitual behavior may hamper efforts to abstain, especially if the alternative does not carry the same appeal (Gardner et al., 2021).

Pioneering research on habit decay suggests that an old commuting habit may gradually decrease as a new commuting habit gradually increases over 4 weeks after the relocation of the workplace office (Walker et al., 2015). Additionally, implementation intentions have been shown to decrease habit strength of smoking (Armitage, 2016) and to replace old waste disposal habits with recycling (Holland et al., 2006). Although these studies provide insights about the conditions that may facilitate habit decay, they do not inform about *how* habit decay may occur over time in daily life at a granular level.

### Temporal trajectories of habit strength in daily life

In the absence of research on how habit decay occurs over time, findings from “habit formation tracking studies” (Gardner et al., 2022, p. 3) can roughly serve to inform how this process may occur and what methods are suitable for this field of research. To our knowledge, five studies have examined the temporal trajectory of habit formation with intensive longitudinal data over a period of several months (Baretta et al., 2024; Fournier et al., 2017; Keller et al., 2021; Lally et al., 2010; Van Der Weiden et al., 2020). Across these studies, participants were instructed to establish a new habit by repeating a novel behavior (predominantly nutrition‐ or physical activity‐related) once per day in response to a specified cue for approximately 3 months, where habit strength was recurringly assessed with the Self‐Report Habit Index (SRHI) (Verplanken & Orbell, 2003) or its subscale, the Self‐Report Behavioral Automaticity Index (SRBAI) (Gardner et al., 2012). Findings coherently suggest that successful habit formation may be described by a non‐linear increasing trend, where the rate of change gradually slows down as the habit strength approaches an upper bound (Fournier et al., 2017; Keller et al., 2021; Lally et al., 2010; Van Der Weiden et al., 2020). Furthermore, findings suggest that the change in habit strength over time varies considerably between individuals, where consistent performance of the novel behavior in response to encountering the cue is key for habit formation to occur (Baretta et al., 2024; Keller et al., 2021; Lally et al., 2010). Based on these studies, the time needed for habit formation to occur has been estimated to range from a matter of days to almost 1 year, with potentially only a minority of participants succeeding in forming a habit (Fournier et al., 2017; Keller et al., 2021; Lally et al., 2010). Noteworthily, estimates of long duration (e.g. 335 days) (Keller et al., 2021) are based on extrapolated predictions beyond the observed time frame.

In terms of modelling the process of change, important commonalities and differences across the studies can be identified. The change in habit strength has been modelled by participants' individual trajectories (Lally et al., 2010), by group‐level modelling (Fournier et al., 2017; Van Der Weiden et al., 2020), or by combining both approaches (Baretta et al., 2024; Keller et al., 2021). Modelling person‐specific trajectories has the advantage of highlighting the idiosyncratic nature of habit formation (Keller et al., 2021; Lally et al., 2010), but research questions related to group differences in habit change processes may be more appropriately addressed with group‐level modelling (Fournier et al., 2017; Keller et al., 2021). The specific models used for describing change over time include non‐linear models estimating an upper asymptote (i.e. an upper bound), including a power curve (Lally et al., 2010), an asymptotic model (Baretta et al., 2024; Keller et al., 2021), and a logistic model (Fournier et al., 2017), as well as the quadratic model (Keller et al., 2021; Van Der Weiden et al., 2020). Taken together, no single model seems suitable for predicting habit strength over time across all participants (Keller et al., 2021; Lally et al., 2010). While asymptotic models have the strength of including parameters with meaningful interpretation—particularly the upper asymptote allowing for estimation of time for habit formation to occur (Lally et al., 2010), the quadratic model is more flexible in modelling diverse arrays of habit strength trajectories, including discontinued habit formation (Keller et al., 2021). More recently, machine learning methods and objective behavioral data have been used to model habit formation over time based on context‐related predictability (Buyalskaya et al., 2023). Concordant with self‐report studies, these findings suggest habit strength to increase asymptotically, with large differences evident between individuals. To conclude, based on the described habit formation research, it is reasonable to assume that habit decay varies considerably between individuals, multiple models may be required to describe this change process across a sample, and that idiographic and nomothetic approaches may provide complementary insights.

### The present study

This study seeks to extend research by investigating the trajectory of habit decay in daily life. We will do so at the example of four health‐risk behaviors to explore whether the temporal trajectories are similar or distinct across behaviors. We chose the four key health‐risk behaviors that contribute to non‐communicable disease (Habib & Saha, 2010; Keeney, 2008; Muller et al., 2016) as examples, that is, sedentary behavior, unhealthy snacking, alcohol consumption, and tobacco smoking. The first aim of the study was to describe how habit strength trajectories change in individuals who set out to degrade a habit. To this end, we fit six models (constant, linear, quadratic, cubic, asymptotic, and logistic) to investigate which models display the best fit with person‐specific and group‐level models. Person‐specific models were further used to estimate how long it takes for habit decay to stabilize. The second aim of the study was to examine, based on group‐level modelling, whether habit decay trajectories differ between the four health‐risk behaviors. The coregistration (Benning et al., 2019) of the study is available online (https://osf.io/g6wpr).

## METHODS

This study reports the primary findings from an online‐based 91‐day intensive longitudinal study with four parallel non‐randomized groups, that is, one of four behaviors that participants self‐selected to change: sedentary behavior, unhealthy snacking, alcohol consumption, or tobacco smoking.

### Population and sample

Eligibility criteria for participation were age of at least 18 years and willingness to reduce one of the four health‐risk behaviors. Participants were excluded if they were not fluent in German, did not own an iOS or Android smartphone, or did not provide informed consent. Additionally, participants were required to respond to the Day 7 survey to proceed with study participation. Participants were retained for analyses if they provided at least six habit strength (SRBAI) measurements to ensure sufficient data (Keller et al., 2021) and engaged with the study at least until the midpoint of the habit decay phase (Day 48) to ensure sufficiently long duration of participation.

Due to a lack of reliable estimates of required parameters, a priori power analysis was not conducted. The planned sample size of 200 (*n* = 50 per behavioral group) was based on a rule of thumb for achieving power of .80 (alpha = .05) in a two‐level model (observations nested within individuals) to detect a small effect size of time and a medium effect size for between‐person differences, assuming a large intraclass correlation coefficient (Arend & Schäfer, 2019).

### Measures

The primary outcome of interest (habit strength) was measured with the 4‐item SRBAI (Gardner et al., 2012) and adapted from the German translation (Verplanken, 2007). The SRBAI assesses the *perception* of habit‐related automaticity (Gardner & Lally, 2023; Orbell & Verplanken, 2015). The SRBAI was phrased to reference performing the target behavior in response to the cue selected by the participant (in brackets), such as “Smoking in this situation (drinking coffee in the morning) is something …” followed by four statements scored on a 5‐point Likert scale, for example, “… that I do automatically.” Items were scored from 0 to 4, with higher scores indicating stronger agreement. The overall SRBAI score was calculated by averaging the four items. Habit strength was measured daily starting from Day 7 (after participants had defined their cue) until study completion (Day 91). In the current dataset, the SRBAI displayed high reliability for between‐person averages (*R*_*KF*_ ≈ 1) and to detect within‐person change (*R*_*C*_ = 0.86) (Cranford et al., 2006; Keller et al., 2021).

While studies have convincingly shown that individuals can reflect on their habits (Orbell & Verplanken, 2010), tracking change in habit based on the perception of habit strength (SRHI/SRBAI) has also received criticism. The case has been made that such self‐report may mirror cue‐behavior performance (Sniehotta & Presseau, 2012), where for example the perception of habit strength could decrease because of not experiencing the relevant cue (Keller et al., 2021). On the contrary, it is reasonable to assume that an individual with a strong habit can reflect on their perceived automaticity, and the noted validity concerns are inherent to all self‐report measurements of mental processes (Orbell & Verplanken, 2015). In acknowledgement of this controversy, exploration of habit strength trajectories in relation to cue‐behavior performance was conducted with data visualization (see Supporting Information S1: Section 2.4).

At baseline, several interindividual difference measures were assessed. The following constructs will be used as sample descriptors in this study and are not outcomes. Target behavior performance prior to baseline was measured using frequency measures for each of the four health‐risk behaviors. Time spent sedentary for the past week was measured with the International Physical Activity Questionnaire short format (Craig et al., 2003). Past unhealthy snacking, alcohol consumption, and tobacco smoking frequency were assessed by asking participants to report on how many days in a typical week these products are consumed and to estimate how many servings/units are consumed on such days. Intention (De Bruijn et al., 2012) to change the selected habitual behavior was assessed on Day 7 with two items (scored 0–4) that were averaged. See Supporting Information S1: Section 1.1 for further details on item content.

### Procedure

Recruitment was primarily conducted with social media advertisements (one for each behavior), noting habit decay as the focal point of the study. Data collection took place in Switzerland between September 2022 and April 2023. Study participation lasted for 91 days, during which participants received daily end‐of‐day e‐diary questionnaires (via SMS including link to questionnaire). Upon beginning participation, participants were informed about the definition of habit and the procedural phases of the study. During the first 7 days, participants were instructed to observe and report cues experienced that trigger instigation of their target health risk behavior. On Day 7, participants were instructed to select one cue in response to which they wanted to change their habitual response for the following 84 days. Subsequently, participants were introduced to the three alternative strategies for disrupting a habit (substitution, inhibition, and discontinuation; see Supporting Information S1: Section 1.2 for instructions provided to participants). Substitution constitutes replacing an old habit by pairing a new behavioral response to the cue that generates the existing habitual response (Gardner et al., 2021; Gardner & Lally, 2018). Inhibition refers to the willful inhibition of enacting the habitual behavior after the habit impulse has been triggered (Gardner et al., 2021; Quinn et al., 2010). Discontinuation refers to the avoidance of encountering the cue that triggers an impulse towards the habitual behavior, eliminating the possibility of the habit impulse from being activated (Gardner et al., 2021; Walker et al., 2015). Participants were instructed to choose one strategy and guided to formulate an implementation intention (Gollwitzer, 1993), also known as an if‐then plan, according to the selected strategy. Specifically, participants were instructed to formulate a plan linking their selected cue (if‐component) to a desired response (then‐component); for example, “If I am watching TV in the evening, then I will eat grapes.” Of note, for the discontinuation strategy, the if‐then plan instructions guided to avoid future cue encounters, for example: “If I arrive home tonight, then I will remove all snacks from my home.” On Day 48, participants had the opportunity to update the strategy and then‐part of their implementation intention to support the continued viability of plans (this did not impact results, see analysis in the Supporting Information S1: Section 2.3.1). At completion, participants were reimbursed (100 CHF for full study, or 7 CHF per week if participation was shorter).

#### Analyses and preprocessing

Data analyses were conducted with R Statistical Software version 4.1.2 (R Core Team, 2021). The online end‐of‐day e‐diary questionnaire assessing habit strength was accessible throughout the study period. Responses given between times 0:00 a.m. and 12:00 p.m. were recoded to refer to the previous day. Duplicate e‐diary responses for the same day were removed by averaging. No partial responses to the SRBAI scale were evident. SRBAI observations were missing for non‐consecutive days and for longer sequences of days (henceforth referred to as missing gaps). Long missing gaps were more prominent in the second half of the time series. Non‐consecutive missing observations were imputed (see Supporting Information S1: Section 1.3), and long missing gaps retained as such. For details on divergence from protocol, see Supporting Information S1: Section 1.6.

#### Person‐specific models

To examine how habit decay occurs at the person‐specific level when trying to degrade a habit, habit strength (dependent variable) was modelled over time (independent variable) for each participant with six prespecified models of interest: constant, linear, quadratic, cubic, asymptotic, and logistic (see Supporting Information S1: Section 1.4.1 for equations and example plots). The models of interest are derived from previous studies examining habit strength trajectories of habit formation (Fournier et al., 2017; Keller et al., 2021; Lally et al., 2010), with the exception of the cubic model, which was a novel addition. The cubic model parameters and flexibility are similar to the quadratic and additionally estimate a second bend in the trajectory. In order to univocally describe the shape of the temporal trajectory of habit strength, the autoregressive parameter of habit strength was not included in the models.

The best fitting model for each participant was determined by the lowest Bayesian Information Criterion (BIC) index. The BIC index was used to prioritize correct model selection, as opposed to the Akaike Information Criterion, which is better suited for predicting future observations (Chakrabarti & Ghosh, 2011). Person‐specific time series and fitted values were visually inspected to assess model properties and potential misfits (Wagenmakers et al., 2021). Visual inspection indicated substantial variation in how well fitted values described the observed habit strength trajectories. Accordingly, a 4‐step procedure was devised to identify whether the model fitted values provided a valid description of the habit trajectory (henceforth referred to as *valid fitted values*). This procedure entailed (1) identifying decreasing trajectories, where (2) model selection is not impacted by individual observations, (3) the model root‐mean‐square‐error is ≤0.33, and (4) the time series do not contain missing gaps of observations longer than 21 days in length. For further details about this procedure, see Supporting Information S1: Section 1.4.2.

Subsequently, for cases where fitted values were deemed valid, time for habit decay to stabilize was calculated in two ways. For time series where the best fitting model was either the asymptotic or logistic, time for habit decay to stabilize was calculated as time needed for 95% of the (lower) asymptote to be reached (Fournier et al., 2017; Keller et al., 2021; Lally et al., 2010). For time series where the best fitting model was either linear, quadratic, or cubic, a tentative indication of stabilization was operationalized as time until the overall change in habit strength over a 7‐day period was smaller than 0.1 (explorative operationalization not stated in coregistration). A decreasing trend in habit strength that crossed the scale midpoint (before stabilization) was taken as an indication that a substantial change in habit strength is likely to have occurred, as similarly done in habit formation research (Keller et al., 2021). Time for habit decay to stabilize was estimated exclusively within the observed time series (no extrapolation).

#### Group‐level models

The same six previously specified models (except the logistic model; removed because of issues with fitting the model) were used in multilevel modeling. Multilevel modelling is a well‐suited approach for dealing with the nested data structure (here daily habit strength observations nested within participants) and data dependencies. Multilevel modelling allows for estimation of the average trajectory (fixed effects) and variation across individuals (random effects) (Peugh, 2010). Here time was rescaled (varying from 0 to 1.72) to improve model convergence. Random intercepts and slopes were added to the models iteratively (from lower order to higher order where applicable), and the best‐fitting multilevel model was determined with the BIC index. Differences in habit decay between the four health‐risk groups were inspected in two ways: (1) by adding group‐level random effects and (2) by adding interaction terms (separately) to the best fitting multilevel models.

## RESULTS

A total of 194 participants were retained for analyses. Participants retained for analysis and those excluded did not differ based on sociodemographic characteristics. See Supporting Information S1: Section 2.1 for flow diagram of participant retention and information on comparison between the retained and excluded samples. For analyzed participants, the total number of missing SRBAI values across participants included for analysis was 4723 (29%; out of maximum 16,296). Imputation substituted one quarter of all missing SRBAI values (*k* = 1229) across all participants, with on average 6.3 values (range 0–20) being imputed for each participant. There was not a significant difference in percentage of missing values or longest missing gap between the behavioral groups. For more descriptive information on missing SRBAI values, see Supporting Information S1: Section 2.2.

### Sociodemographic and baseline characteristics

The sociodemographic characteristics of the sample retained for analysis (*n* = 194) are displayed in Table 1 (for comprehensive baseline characteristics, see Supporting Information S1: Section 2.1). Overall, the median age of the sample was 39 (interquartile range 32–49) and predominantly identified with female gender (75%). The majority of the sample had completed vocational training or university studies (87%) and were employed (80%). Post‐hoc pairwise behavioral group comparison on baseline characteristics revealed that the sedentary behavior and unhealthy snacking groups were younger and had a higher proportion of women than the alcohol consumption group. Likewise, the sedentary behavior group was significantly younger than the smoking group.

**TABLE 1:** Sample characteristics by behavioral group (*N* = 194).

	*N*	Overall	SB, *N* = 46	US, *N* = 57	AC, *N* = 52	TS, *N* = 39	*p*
Baseline variables							
Age	194	39 (32, 49)	34 (30, 42)	38 (30, 45)	44 (36, 53)	40 (34, 51)	<.001
Gender	183						<.001
Female		137 (75%)	37 (84%)	49 (89%)	24 (51%)	27 (73%)	
Male		45 (25%)	6 (14%)	6 (11%)	23 (49%)	10 (27%)	
Other		1 (0.5%)	1 (2.3%)	0 (0%)	0 (0%)	0 (0%)	
BMI	183	23.5 (21.8, 27.5)	22.1 (20.8, 25.3)	24.8 (21.9, 27.9)	24.7 (22.0, 27.9)	23.5 (22.0, 26.6)	.109
Day 7 variables							
SRBAI	194	3.00 (2.25, 3.50)	3.63 (3.25, 4.00)	2.50 (2.25, 3.25)	2.25 (1.69, 2.75)	3.25 (3.00, 3.75)	<.001
Intention	194	3.50 (3.00, 4.00)	3.25 (3.00, 4.00)	3.00 (3.00, 4.00)	3.00 (3.00, 4.00)	4.00 (3.00, 4.00)	.367
Decay strategy	194						<.001
Substitution		113 (58%)	36 (78%)	32 (56%)	34 (65%)	11 (28%)	
Inhibition		68 (35%)	7 (15%)	23 (40%)	14 (27%)	24 (62%)	
Discontinuation		13 (6.7%)	3 (6.5%)	2 (3.5%)	4 (7.7%)	4 (10%)	

*Note*: Continuous variables reported with median (interquartile range) and *p*‐values based on Kruskal–Wallis rank sum test; categorical variables reported with *n* (%) and *p*‐values based on Fisher's exact test for count data with simulated *p*‐value (based on 2000 replicates).

Abbreviations: AC, alcohol consumption; BMI, body mass index; SB, sedentary behavior; SRBAI, Self‐Report Behavioral Automaticity Index; TS, tobacco smoking; US, unhealthy snacking.

The sedentary behavior group spent a median of 9 h sedentary per day (interquartile range 7–10 h; *n* = 46). The unhealthy snacking group ate a median of 14 unhealthy snack portions per week (interquartile range 10–21 portions; *n* = 56). In the alcohol consumption group, the median number of units of alcohol per week was 12 (interquartile range 8–29 portions; *n* = 52). The smoking group consumed a median of 105 tobacco product units (e.g. cigarettes) per week (interquartile range 70–140 cigarettes; *n* = 39).

Based on the Day 7 survey, there was a group difference in initial habit strength, whereby the sedentary behavior and smoking groups both displayed significantly stronger habits than both alcohol and snacking groups. Participants reported having strong intention towards reducing their habitual target behavior in response to encountering their cue. Overall, the most commonly selected habit decay strategy (based on multiple choice selection) was substitution (58%, *n* = 113), followed by inhibition (35%, *n* = 68). Here, the smoking group differed from the sedentary and alcohol groups. Substitution was clearly preferred in the sedentary behavior (78%, *n* = 36) and alcohol consumption (65%, *n* = 34) groups, followed by inhibition (15% and 27%, respectively). In the smoking group, inhibition was selected most often (62%, *n* = 24), followed by substitution (28%, *n* = 11). The cues selected by participants referred to physical and social contexts, emotions/cognitions, and events/temporal contexts (see Supporting Information S1: Section 2.1).

### Person‐specific habit decay models

All six models of interest converged for all 194 participants in person‐specific modelling. Based on the BIC index, the asymptotic and logistic models were the most common best fitting models, followed by the polynomial cubic and quadratic models, with the constant and linear models being least frequently the best fitting models. There was no significant difference in the distribution of best‐fitting models by behavioral group (see Table S4: Section 2.3). For descriptive statistics on best‐fitting person‐specific models and related sensitivity analyses, see Supporting Information S1: Section 2.3. The sensitivity analyses largely confirmed results.

**FIGURE 1:**
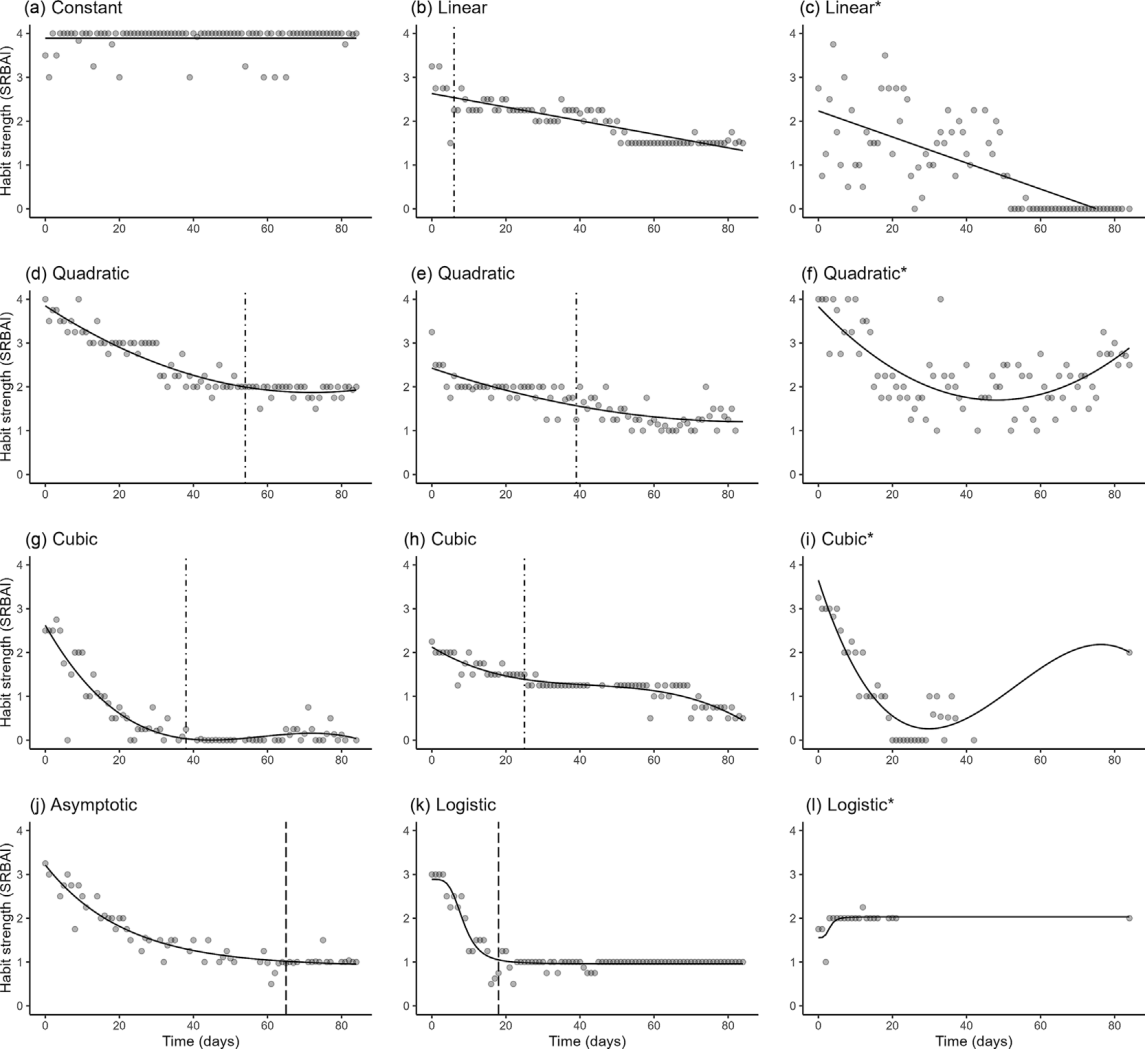
Examples of person‐specific time series with best‐fitting constant, linear, quadratic, cubic, asymptotic, and logistic models (*N* = 12). Note: SRBAI = Self‐Report Behavioral Automaticity Index; grey points = observations; black lines = model fitted values; dot‐dashed vertical lines = first occurrence when change in model fitted values is <.01 during 7‐day period (b: 6, d: 54, e: 39, g: 38, h: 25 days); dashed vertical lines = time when 95% of the lower asymptote is reached (j: 65, k: 18 days); *model fitted values deemed nonvalid.

We identified 5 linear (26%), 4 quadratic (17%), 14 cubic (45%), 28 asymptotic (54%), and 32 logistic (60%) best‐fitting models to have valid fitted values of the habit trajectory. Regarding the constant model, predicting habit to be stable over time was deemed valid for six participants (43%). See Figure 1 for examples of person‐specific model plots.

Time for habit decay to stabilize ranged from 1 to 65 days (median 9–10 days; *n* = 42) based on asymptotic and logistic model fitted values reaching 95% of the lower asymptote within the observed time series for cases where fitted values crossed the scale midpoint. Comparatively, for linear and polynomial models, a similar range of time needed for habit decay to stabilize was observed (see Table 2).

**TABLE 2:** Median (min, max) estimates of time in days for habit strength to decrease below scale midpoint and alternative definitions for habit decay to stabilize by best fitting models (*N* = 60^a^).

Operationalization	Asymptotic (*n* = 21)	Logistic (*n* = 25)	Linear (*n* = 3)	Quadratic (*n* = 4)	Cubic (*n* = 7)
Decreases below scale midpoint	2 (1, 27)	2 (1, 54)	62 (41, 79)	14.5 (8, 54)	5 (1, 16)
Stabilizes^b^ for 7 days	19 (7, 51)	11 (6, 29)	6 (6, 6)	41.5 (6, 54)	30 (21, 38)
Reaches 95% of lower asymptote	10 (1, 65)^c^	9 (3, 52)^d^	N/A	N/A	N/A

*Note*: Estimates are based on best‐fitting person‐specific model predictions with (1) decreasing trends, (2) stable model selection, (3) RMSE value ≤0.33, (4) missing gaps of observations ≤21 days, and (5) predictions cross the value 2.

Abbreviation: N/A, not applicable.

^a^ Sample size based on described selection procedure.

^b^ Stabilization defined by first occurrence when change in model fitted values is <.01 during 7‐day period.

^c^ *n* = 19.

^d^ *n* = 23.

Because this was the first intensive‐longitudinal study of habit decay, we explored how habit strength varied in relation to cue‐behavior performance. This exploration suggested that habit strength is distinct from cue‐behavior performance and that there is an idiosyncratic association between the two. The visual analyses shown in Supporting Information S1: Section 2.4 show that for some participants, habit gradually decreased while the behavior was consistently not performed at cue encounter. Other participants showed no changes in habit strength at no performance or less visible associations between cue‐behavior performance and habit strength.

### Group‐level modelling

**FIGURE 2:**
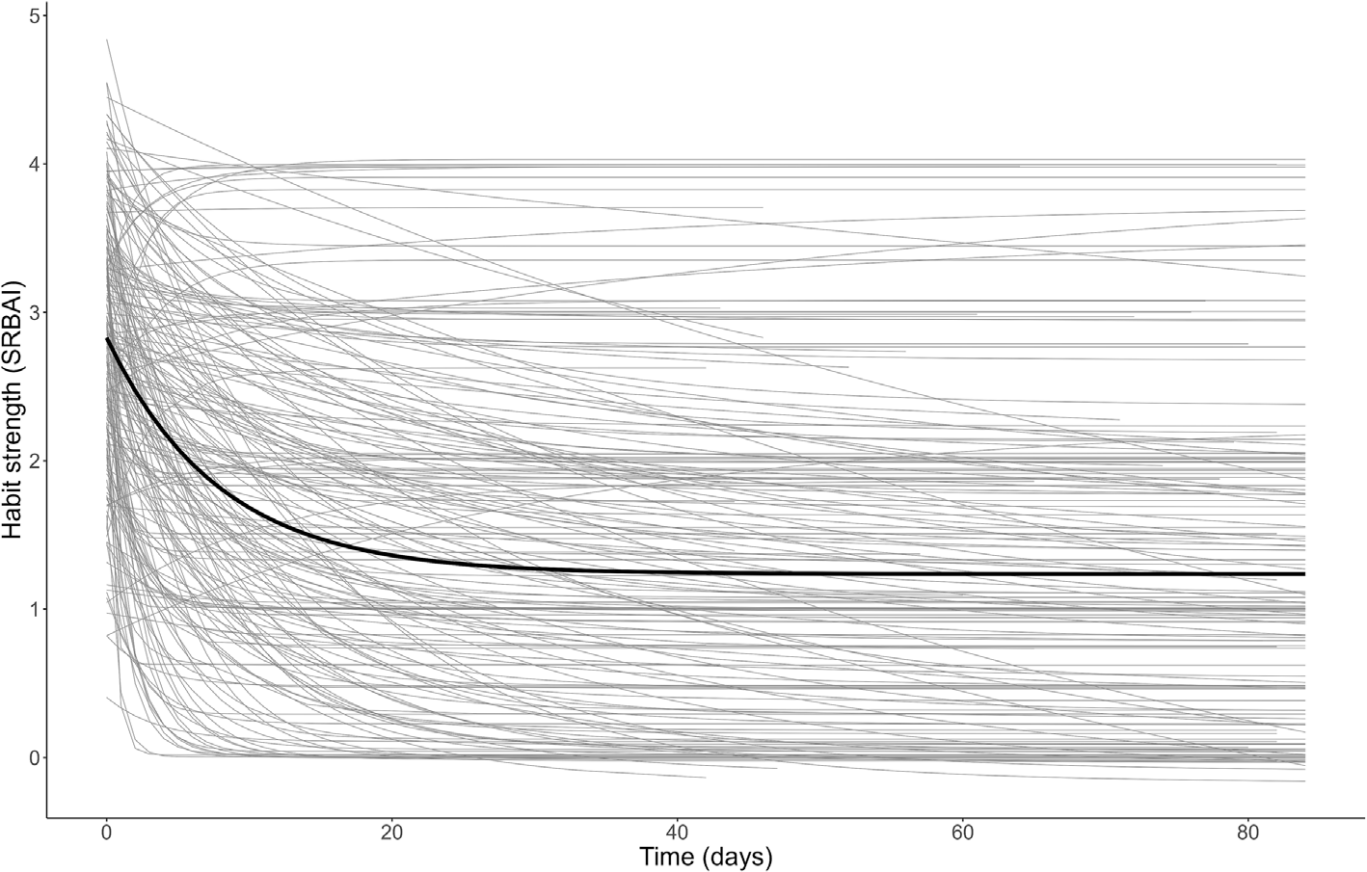
Asymptotic multilevel model predicting habit strength by time (*N* = 194). Note: The black line represents the fixed effect fitted values, and the grey lines represent person‐specific fitted values. SRBAI = Self‐Report Behavioral Automaticity Index.

The intraclass correlation coefficient of the constant model suggested 76% of variance was due to between‐person differences. The model displaying the best fit was the asymptotic model with between‐person random effects estimated for all parameters. Results indicated that on average habit strength decreased with a decelerating trend, with substantial between‐person variance evident (see Figure 2 below and Table S10: Section 2.5.1). Estimation issues were encountered with the asymptotic model, suggesting challenges in obtaining accurate standard errors and confidence intervals. The second best‐fitting multilevel model was the cubic with random effects estimated for all parameters, and no estimation issues were encountered. The curves of the asymptotic and cubic model fixed effects were similar in shape (see Figure S4: Section 2.5).

**FIGURE 3:**
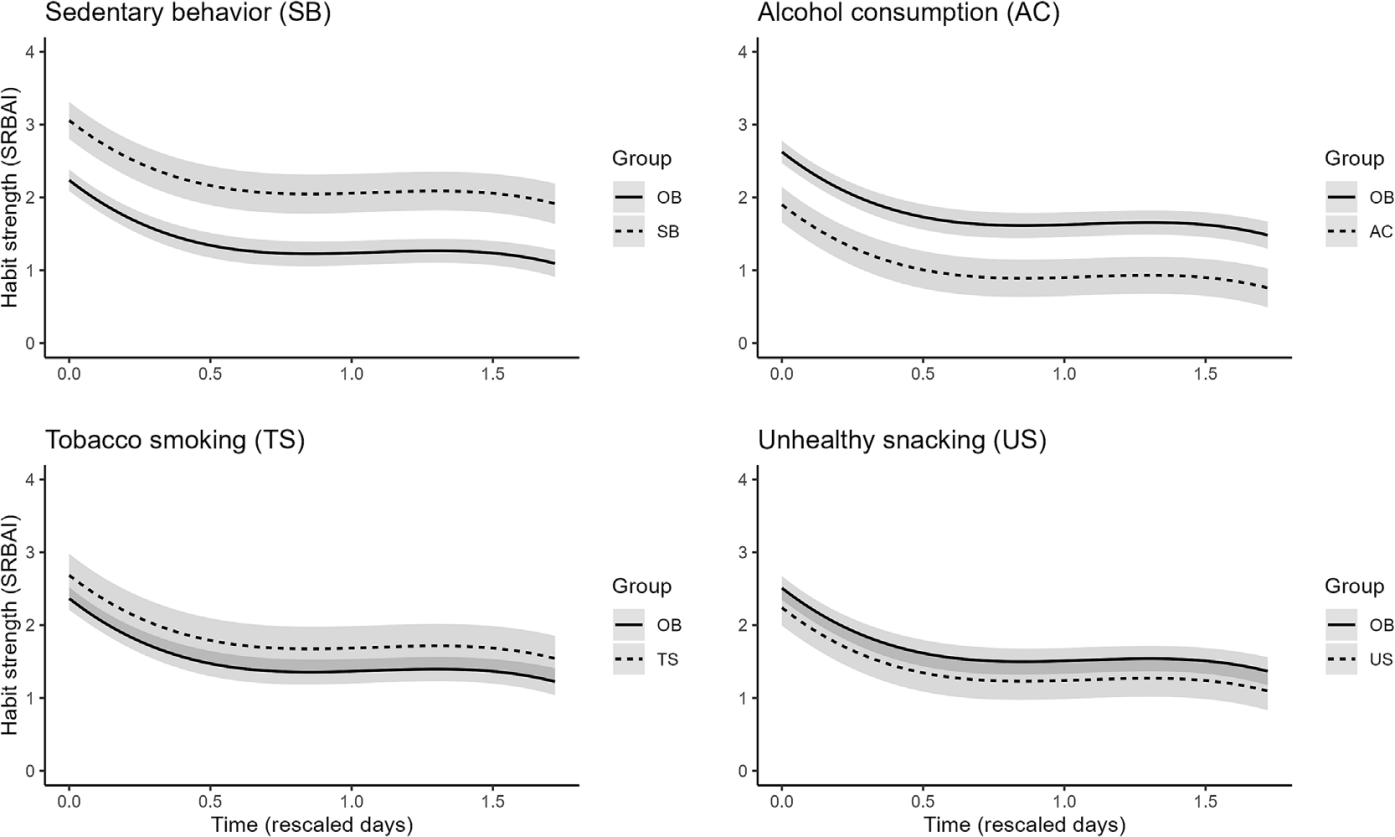
Multilevel cubic model plots with main effect for behavioral group (*N* = 194). Note: OB = other behaviors average effect; SRBAI = Self‐Report Behavioral Automaticity Index. Time (Days 0–84) has been rescaled to vary from 0 to 1.72.

To address behavioral group differences, the asymptotic and cubic models were both used. First, behavioral group differences were investigated by adding random effects for the behavioral groups to the asymptotic model. Indicating group differences, the results suggested that allowing for group variation in initial habit strength improved model fit (see Table S10: Section 2.5.1). Second, group differences in the habit decay trajectory were investigated by adding interaction terms. For this analysis, we used the cubic instead of the asymptotic model due to convergence issues and limited capacity to modify parameters with the latter. We added main effects of behavioral group and interaction terms of behavioral group and time to the model. Separate multilevel models were run for each behavior, accounting for behavioral groups with dichotomous variables indicating group membership. The models showed no indication of differences in habit trajectories between the four behavioral groups. However, findings corroborated the results of the asymptotic model in that model fit improved when allowing the intercept to vary by behavioral group. Cubic model parameters indicated that initial habit strength was higher for sedentary behavior ($\beta_{0}$ = 3.06, CI_95_ range 2.77–3.35) and lower for alcohol consumption ($\beta_{0}$ = 1.90, CI_95_ range 1.62–2.17) compared with the combined averages of the other behavior groups ($\beta_{0}$ = 2.24, CI_95_ range 2.09–2.38 and $\beta_{0}$ = 2.62, CI_95_ range 2.47–2.78, respectively). See Figure 3 plots for depiction of behavior group differences. Of note, cubic model diagnostics suggested residuals to be autocorrelated. See Supporting Information S1: Sections 2.5.1–2 for further information on multilevel modelling results and related sensitivity analyses. Sensitivity analyses largely confirmed results.

## DISCUSSION

This study investigated the trajectories of habit decay in real‐world settings of people who tried to degrade a habit at the example of four health‐risk behaviors. Based both on person‐specific and multilevel models, habit decay is often a process where the initial decrease in habit strength is faster and gradually approaches a steady state after some time. Multilevel analysis of behavioral group differences suggested that initial habit strength may be higher for sedentary behavior and lower for alcohol consumption. Behavioral group differences in the trajectory of habit decay were not identified, with results rather highlighting the idiosyncratic nature of this process.

### How does habit strength decrease over time?

Present findings are in line with habit formation research suggesting change in habit strength to be typically described by non‐linear processes of change with pronounced interindividual differences (Fournier et al., 2017; Keller et al., 2021; Lally et al., 2010; Van Der Weiden et al., 2020). These observed interindividual differences are subsequently described. First, habit strength may decrease in a decelerating fashion such that it ultimately reaches a newfound stable state. This is evident and explicitly estimated by asymptotic and logistic models (see Figure 1j,k). Furthermore, this was tentatively visible for some polynomial models (see Figure 1d,e,g), as comparatively concluded in the context of habit formation (Keller et al., 2021). When decay and stabilization occur rapidly, this may be termed habit disruption. Second, habit strength may decrease without clear stabilization at a lower bound (see Figure 1b,h) based on linear and polynomial model estimates. Third, findings show that the lower bound that habit strength approaches may differ between individuals (e.g. comparing Figure 1d,h,j).

Idiographic modelling also showed that habit decay may be unsuccessful in various ways. First, in some instances, a habit retained its strength over time (e.g. Figure 1a), which can be described as a stable, strong habit (Rebar et al., 2022). Second, in some instances, habit gained strength over time (e.g. Figure 1l). Habit strengthening may be an indication of relapse to a habitual tendency that precedes the observed time series. Third, in some instances, habit strength initially decreased but subsequently regained strength (see Figure 1f,i). This discontinued change process is captured by a U‐shaped curve of polynomial models, as also comparatively suggested in the context of habit formation (Keller et al., 2021).

### How long does it take for habit decay to stabilize?

Based on the time needed for 95% of the lower asymptote to be reached within the observational period (*n* = 42; 22% of sample), habit decay stabilization typically took under 2 weeks, ranging from 1 to 65 days (see Figure 1j,k). Regardless of whether habit decay is better described by the asymptotic or logistic model, habit strength reaches the lower bound within similar periods of time (Table 2). Estimates of time for habit decay to stabilize based on linear and polynomial models were similar, although interpretation warrants caution as the operationalization is less stringent. For example, estimating the time for habit decay to stabilize based on when the change in habit strength during a 7‐day period is less than 0.1 seems to perform well when habit strength initially decreases rapidly and later slows down (see Figure 1d,g), but less optimally when the decrease is more gradual (Figure 1b,e) or includes a transient phase of stabilization (Figure 1h).

Interpretation of findings indicating short duration of habit decay stabilization (e.g. 1 day) warrants caution. This very short duration is not in line with habit theory, which suggests that habits take time to change. Potentially, this short duration is linked to issues of measurement validity (see Section 4.5). Also, decay stabilization does not necessitate that the habit has been “broken.” It is further important to note that a comparison of durations in habit decay and habit formation processes is difficult due to differences in procedures. Habit formation studies have reported a median of approximately 60 or more days for habits to form (Keller et al., 2021; Lally et al., 2010). However, habit formation studies utilized different procedures, including constraining cue‐behavior repetition to once per day and extrapolation of model predictions beyond the observed time series.

### Behavioral differences

Multilevel modelling highlighted that substantial variance in habit decay trajectories is largely due to differences between individuals and that this between‐person heterogeneity is larger than target behavior‐related differences. Nonetheless, multilevel modelling results indicated that habits for sedentary behavior exhibit greater initial strength and that habits for alcohol consumption exhibit lower initial strength compared with the other behaviors studied (see Figure 3), as also indicated by baseline behavioral group comparisons (see Table 1). These differences in initial habit strength may reflect random variation in sampling or a systematic difference between the target behaviors. It could be that the stronger initial habit observed for sedentary behavior is due to a higher frequency of cue‐behavior repetition in the past. As similarly discussed in the context of the relative ease in establishing hand‐washing habits (Buyalskaya et al., 2023), present findings of strong initial habit strength for sedentary behavior may be linked to the fact that this behavior is frequently enacted and part of chunked action sequences (Balleine & Dezfouli, 2019).

### Strengths and limitations

The present study bears several strengths in terms of procedures and data analysis. Importantly, this is the first study to investigate habit decay in daily life with intensive longitudinal data. Sampling from the general population, intensive longitudinal assessment, and allowing participants to select personally relevant cues informed by an observational period enabled ecologically valid investigation. In terms of statistical practice, extensive data visualization and diverse modelling approaches align with practices promoting transparency and acknowledgment of uncertainty (Wagenmakers et al., 2021).

The study naturally also has limitations. First, measuring the perception of automaticity as an indicator of habit strength is constrained because habit operates outside of conscious awareness (Gardner & Tang, 2014; Hagger et al., 2015). Second, although sampled from the general population, the convenience sample consisted of highly educated individuals with strong intention towards breaking their habit, which sets limitations to the generalizability of results. Third, the procedures to identify model‐fitted values as valid used cut‐off scores. While these cut‐offs are arbitrary and do not perform optimally across all trajectories, this approach allowed for systematic classification not prone to subjective bias. Fourth, multilevel modelling was restricted by estimation issues. The logistic model was not used in multilevel modelling, and the asymptotic model could not be leveraged to its full potential, which is attributable to the large heterogeneity in trajectories.

### Future directions

The present study can inform future habit research on what to expect regarding data quality and how to potentially handle and interpret similar intensive longitudinal data. Findings demonstrate that idiographic approaches provide a more detailed understanding of the heterogeneous habit change processes compared with nomothetic approaches. Additionally, the study illustrates how accounting for missing gaps (see Figure 1i,l) and model absolute fit (see Figure 1c,f) play complementary roles in determining model accuracy, providing insights into the boundaries of intensive longitudinal modelling and the confidence in evidence of such modelling efforts. Findings also highlight how isolated observations following a long missing gap may impact model selection and lead to potentially inaccurate models (see Figure 1i). Relatedly, we posit that estimation of time for habit decay to stabilize strictly within the observed time series provides a stronger evidence base compared with incorporating extrapolation of model predictions.

This study provided initial evidence for the credibility of habit strength self‐report measurement in the context of habit degradation by showing that habit strength is distinct from cue‐behavior performance. For example, a self‐reported habit can change gradually (and not abruptly) when not performing the behavior at the occurrence of the cue (see Supporting Information S1: Section 2.4). While our exploratory findings are encouraging, more studies are needed to unveil the boundary conditions for the validity of a self‐reported habit strength measurement in the context of habit decay. Habit strength being distinct from cue‐behavior performance does not rule out the potential influence of relevant experiences such as habitual behavioral slips or a lack of cue encounters (Keller et al., 2021; Sniehotta & Presseau, 2012), which may partially explain habit strength fluctuation (see Figure 1c,f) and the speed with which habit disruption often occurred.

The field of habit research would benefit from more precisely defined criteria for self‐report habit measurements as to what constitutes a habit and substantial change in habit strength. In the present study, meaningful change was (arbitrarily) defined by necessitating habit strength to cross the scale mid‐point, as done previously in habit formation research (Keller et al., 2021; Lally et al., 2010), but this criterion seems insufficient. For example, change amounting to less than a one‐point decrease on the Likert scale (that crosses the scale mid‐point) may be appropriately described as a relatively stable process over time (see Figure 1l). Future research should strive to observe habit decay in daily life beyond 3 months to capture how this process may be maintained over extended periods of time.

There are some preliminary recommendations for intervention studies that can be made based on the habit degradation strategies participants were guided to use. Interventionists are encouraged to leverage the substitution and inhibition strategies, as based on the uptake, these are preferred approaches compared with discontinuation (see Table 1). However, more research is needed to compare the effectiveness of alternative degradation strategies. As such research needs to account for fidelity (e.g. implementation frequency) and cue characteristics (e.g. frequency of encounters), this was beyond the scope of the present paper.

### Conclusions

Investigating habit decay with high resolution elucidates the multitude of potential forms this temporal process may take. For future intensive longitudinal habit research, it is recommended to utilize idiographic approaches with non‐linear models of change and to extensively leverage data visualization. Present findings are moderately encouraging in terms of the viability of degrading unhealthy habits with usage of implementation intentions and hold promise for the development of interventions to overcome unhealthy habitual tendencies.

## CONFLICT OF INTEREST STATEMENT

The authors have no conflicts of interest to declare.

## ETHICS APPROVAL STATEMENT

The Ethics Committee of the Faculty of Human Sciences at the University of Bern has granted ethical approval for the study (Nr. 2021‐11‐00004).

## ADDITIONAL RESOURCES

For central R code, data, and comprehensive data visualizations, see: https://osf.io/sngu4/.

## Supporting information


**Figure S1.** Examples of potential habit strength trajectories.
**Figure S2.** Flow diagram of participant retention.
**Figure S3.** Person specific plots of habit strength and cue‐behavior performance.
**Figure S4.** Plot of two‐level asymptotic and cubic model fixed effects.
**Figure S5.** Multilevel cubic model plots with day 7 intention as covariate.
**Table S1.** Comprehensive baseline sample characteristics by behavioral group (*N* = 194).
**Table S2.** Examples of cues selected by participants by behavioral group.
**Table S3.** Descriptive statistics of missing SRBAI values in time series (*N* = 194) after imputation.
**Table S4.** Frequencies (%) of best fitting person‐specific models across behavioral groups (*N*=194).
**Table S5.** Distribution of RMSE across best‐fitting person specific models (*N*=194).
**Table S6.** Median (interquartile range) of missing value statistics across best fitting person‐specific models (*N* =194).
**Table S7.** Frequencies (%) of best fitting person‐specific models without missing value imputation across behavioral groups (*N*=194).
**Table S8.** Frequencies (%) of how if‐then plan strategies were changed on day 48 (*N* = 59).
**Table S9.** Median (interquartile range) of change in linear slope of habit strength from the week proceeding day 48 to the following week by if‐then plan update status (*N* = 163).
**Table S10.** Comparison of two‐ and three‐level models predicting habit strength with different time parameters (*N* = 194).
**Table S11.** Multilevel cubic models with behavioral group main effects (*N* = 194).
**Table S12.** Fixed effect estimates [95% confidence interval] for multilevel cubic models with interaction terms for each behavioral group.
**Table S13.** Multilevel cubic model predicting habit strength by time with main effect for behavior group without missing value imputation (*N* = 194).
**Table S14.** Multilevel cubic model predicting habit strength by time with main effect and interaction terms for day 7 intention (*N* = 194).

## Data Availability

Core R‐scripts, data, and comprehensive data visualizations are available on https://osf.io/sngu4/.
