# Supplementary material for: The temporal trajectories of habit decay in daily life: An intensive longitudinal study on four health‐risk behaviors
Source: Appl Psychol Health Well Being. 2024 Oct 25;17(1):e12612. doi: 10.1111/aphw.12612 (PMC11635905; doi:10.1111/aphw.12612)
Supplement: Supplementary file 1 — Figure S1. Examples of potential habit strength trajectories. Figure S2. Flow diagram of participant retention. Figure S3. Person specific plots of habit strength and cue‐behavior performance. Figure S4. Plot of two‐level asymptotic and cubic model fixed effects. Figure S5. Multilevel cubic model plots with day 7 intention as covariate. Table S1. Comprehensive baseline sample characteristics by behavioral group (N = 194). Table S2. Examples of cues selected by participants by behavioral group. Table S3. Descriptive statistics of missing SRBAI values in time series (N = 194) after imputation. Table S4. Frequencies (%) of best fitting person‐specific models across behavioral groups (N=194). Table S5. Distribution of RMSE across best‐fitting person specific models (N=194). Table S6. Median (interquartile range) of missing value statistics across best fitting person‐specific models (N =194). Table S7. Frequencies (%) of best fitting person‐specific models without missing value imputation across behavioral groups (N=194). Table S8. Frequencies (%) of how if‐then plan strategies were changed on day 48 (N = 59). Table S9. Median (interquartile range) of change in linear slope of habit strength from the week proceeding day 48 to the following week by if‐then plan update status (N = 163). Table S10. Comparison of two‐ and three‐level models predicting habit strength with different time parameters (N = 194). Table S11. Multilevel cubic models with behavioral group main effects (N = 194). Table S12. Fixed effect estimates [95% confidence interval] for multilevel cubic models with interaction terms for each behavioral group. Table S13. Multilevel cubic model predicting habit strength by time with main effect for behavior group without missing value imputation (N = 194). Table S14. Multilevel cubic model predicting habit strength by time with main effect and interaction terms for day 7 intention (N = 194). [file APHW-17-0-s001.pdf]

The temporal trajectories of habit decay in daily life: An intensive longitudinal study on four health-risk behaviors – Supplementary file

Authors: Robert Edgren, MSc, Dario Baretta, PhD, & Jennifer Inauen, PhD

Table of Contents

1. Methods 3

1.1 Measures 3

1.1.1 Target behaviors 3

1.1.2 Intention 3

1.2 Procedure 3

1.3 Missing value imputation 6

1.4 Person-specific modelling 6

1.4.1 Model formulae 6

1.4.2 Procedure for identifying "valid fitted values" 8

1.5 Group-level models 9

1.6 Divergence from protocol 9

2. Results 9

2.1 Sample characteristics 9

2.2 Description of missing SRBAI observations 13

2.3 Person-specific modelling 13

2.3.1 Sensitivity analyses 15

2.4. Exploration of habit strength trajectories in relation to cue-behavior performance 16

2.4.1 Methods 17

2.4.2 Results 18

2.4.3 Discussion 20

2.5 Group-level modelling 21

2.5.1 Behavioral group differences 21

2.5.2 Sensitivity analyses 26

List of figures

Figure S1: Examples of potential habit strength trajectories……………………………………. 7

Figure S2: Flow diagram of participant retention……………………………………………. 10

Figure S3: Person specific plots of habit strength and cue-behavior performance ……………. 19

Figure S4: Plot of two-level asymptotic and cubic model fixed effects……………………….. 21

Figure S5: Multilevel cubic model plots with day 7 intention as covariate……………………. 27

1. Methods

1.1 Measures

1.1.1 Target behaviors

Unhealthy snacks were defined as foods consumed between main meals that are high in fat, sugar, and/or salt and low in micronutrients (Evans et al., 2017; Verhoeven et al., 2014) with examples of unhealthy snacks given based on food categories with high fat/sugar content (Kelly et al., 2007) as similarly done previously (Inauen et al., 2016). Participants were instructed to consider 1 serving of unhealthy snacks as approximately 1 handful or 30 grams in weight (FoodDrinkEurope, 2023). One unit of alcohol was described to correspond to 33cl of 5% beer, 13cl of wine or 4cl of 40% liquor (World Health Organization, 2001). For estimating number of tobacco product units consumed participants were instructed to consider cigarette, cigar, and pipe smoking.

1.1.2 Intention

Intention strength was assessed with the items “To what extent do you intend to reduce your [target behavior] when you encounter the selected situation [selected cue]?” and “I plan to reduce my [target behavior] tomorrow in my chosen situation ([selected cue])?”. Items were measured on 5-point Likert scales (scored 0-4, with higher scores indicating stronger intention). Items were averaged to derive one score for the construct.

1.2 Procedure

Exerts of information and instructional texts provided to participants in the unhealthy snacking group on day 7 are subsequently displayed. Note that these exerts are translated from German, and do not cover all information provided to participants (for example, the definition for habit was provided during the first day of participation, and readily available on all subsequent days during

the first week). The content of texts provided to the other behavior groups are the same, but examples are made in reference to the relevant target behavior.

Now it's time for you to choose a snacking habit that you would like to change during the study. First, we would like to explain what a habit is and how to change habitual snacking behavior. Understanding this is important for participation in the study.

Habitual behavior

By "habitual behavior" we mean a behavior that has become automated due to constant repetition when a cue occurs and has therefore become routine. Cues can be objects, people, routines or times, for example. A strong habitual behavior is then performed almost automatically when the cue occurs. In order to break a habitual behavior, it is therefore important to first find the cue.

Here are a few examples:

Anna eats a pastry during her afternoon coffee break. Personal cue: Afternoon coffee break (routine)

Anna sees the cookie tin in her kitchen cupboard and grabs a cookie straight away. Personal cue: cookie tin (object)

At 4 p.m. Anna sits down and eats a cookie. Personal cue: 4 p.m. (time).

[Page break]

Now it's your turn to choose a cue for your snack habit that you want to change. Remember, you should focus on this cue for your snacking habit during the 12-week study period.

Choose a cue...

- **...that you encounter frequently,**
- **...that tempts you to eat unhealthy snacks most of the time**
- **...and for which you feel confident to stop or reduce your snack consumption during the study.**

Below we have listed the cues that you have observed and reported in your diary over the past week. You can now choose a cue that best meets the criteria above (frequent occurrence, usually leads to snacking, you are confident that you can stop/reduce snacking for this cue).

You are also welcome to choose a cue that is not listed here if you can think of one that fits even better. This will be the snack habit you are trying to change during the study.

[Reported cues inserted here]

I select the following cue: [Open response answer field]

[Page break]

Now it's time to decide how you want to reduce your unhealthy snack consumption in your chosen situation "[selected cue inserted here]".

First, take a look at this description of how habits can be changed:

Changing habits

The key to changing a habit lies in the following three steps:

- First, identify the cues that cause the habitual behavior. You have just completed this step!
- Secondly, you need to create a plan for how you want to behave in future when you encounter the cue.
- And thirdly, the plan must be consistently implemented when you encounter the cue.

[Page break]

Now we come to the second step for habit change: you create a plan for how you want to behave in the future when you encounter the cue.

There are different ways to stop or reduce habitual behavior when you encounter the cue. You can...

- ... replace the behavior with something else (substitute action).
- ... prevent yourself from performing the behavior (inhibition).
- ... prevent yourself from encountering the cue (preventing the situation).

Here are a few examples to illustrate this:

Anna wants to change her habit of eating a pastry with her coffee in the cafeteria in the afternoon. She considers 3 ways in which she can achieve this:

- She could plan to eat a fruit instead of a pastry (replacement action), or
- She could plan to think about her goal of not eating a pastry (inhibition), or
- She could plan to stop drinking coffee in the afternoon (prevent situation).

In the following part, you can switch back and forth between the questions to decide on a plan.

The subsequent guidelines were presented in small chunks using multiple choice questions, where the guidelines displayed differed depending on the answer provided previously. This step-wise presentation of instructions (with the possibility to go back-and-forth through the questionnaire) was used to facilitate comprehension and to make an informed decision about how to proceed with study participation.

1.3 Missing value imputation

Non-consecutive missing observations were imputed with the Kalman filter using the R package imputeTS (Moritz & Bartz-Beielstein, 2017). The decision to impute only non-consecutive missing values was a conservative effort to improve data quality. Preliminary testing of imputing longer missing gaps indicated that imputed values tend to gradually stabilize around a given value, mimicking the approach of an asymptote which cannot be assumed to be correct.

1.4 Person-specific modelling

1.4.1 Model formulae

The formulae of the models predicting habit strength (y) by time are presented below. The predictor variable time was rescaled to vary from 0 to 84, where 0 corresponds to the first habit strength measurement (day 7 of study participation) and 84 corresponds to the last day of study participation (day 91).

Constant: $y_{ti} = \beta_{0i} + e_{ti}$

Linear: $y_{ti} = \beta_{0i} + \beta_{1i}(Time_{ti}) + e_{ti}$

Quadratic: $y_{ti} = \beta_{0i} + \beta_{1i}(Time_{ti}) + \beta_{2i}(Time_{ti})^2 + e_{ti}$

Cubic: $y_{ti} = \beta_{0i} + \beta_{1i}(Time_{ti}) + \beta_{2i}(Time_{ti})^2 + \beta_{3i}(Time_{ti})^3 + e_{ti}$

Asymptotic: $y_{ti} = \beta_{ASYMi} + (\beta_{R0i} - \beta_{ASYMi}) * \exp(-\exp(\beta_{LRCi}) * Time_{ti}) + e_{ti}$

Log-logistic: $y_{ti} = \beta_{LASYMi} + \frac{\beta_{UASYMi} - \beta_{LASYMi}}{1 + \exp[\beta_{SMIDi}(\log(Time_{ti}) - \log(\beta_{XMIDi})]} + e_{ti}$

To clarify the asymptotic model parameters, the *ASYM* represents the asymptote (in case of a decreasing trend, the lower bound) of habit strength, *R0* represents the value of habit strength on day 0, and *LRC* represents the natural logarithm of the rate constant at which habit strength

approaches the asymptote. To clarify the log-logistic model parameters, *LASYM* and *UASYM* represent the lower and upper asymptotes, respectively, *SMID* represents the slope at the midpoint between the asymptotes, and *XMID* represents the time at which habit strength reaches the midpoint between the asymptotes (Onofri, 2019). For fitting person-specific asymptotic and logistic models the R package nls.multstart (Padfield & Granville, 2020) was used, as it enables using multiple starting values iteratively. Example plots of the 6 models of interest are displayed in Figure S1.

Figure S1. Examples of potential habit strength trajectories in the context of trying to degrade a habit based on constant, linear, quadratic, cubic, asymptotic, and logistic models. Dashed lines represent trajectories of habits with higher initial strength, less pronounced decrease, or transient decrease in habit strength.

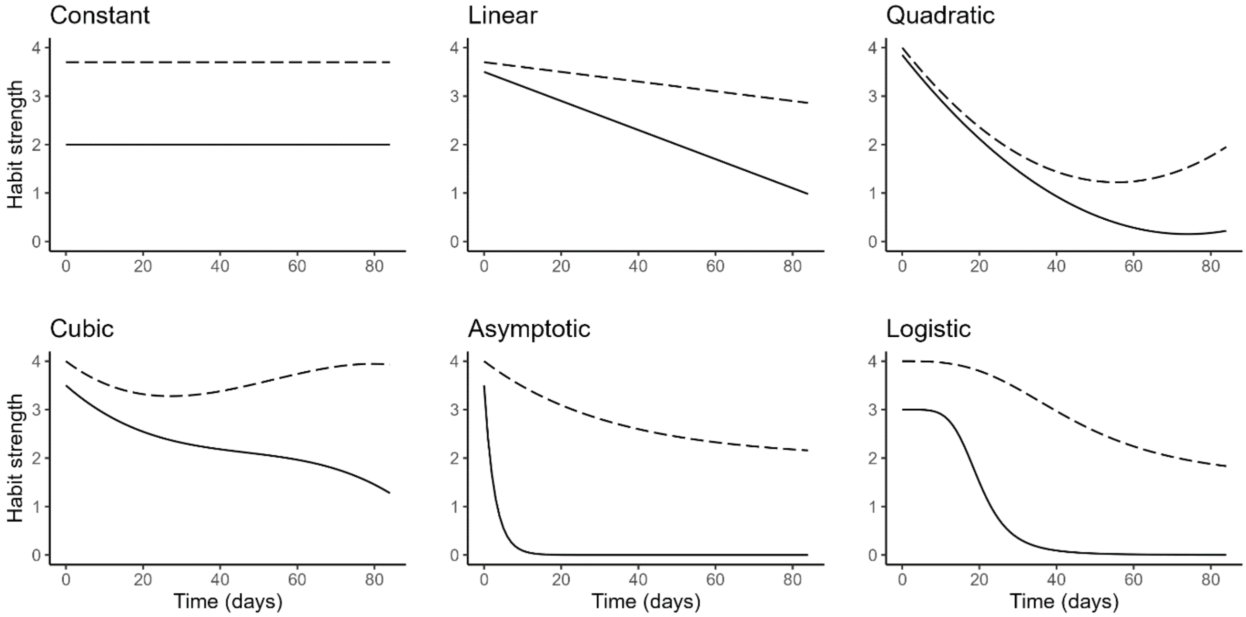

1.4.2 Procedure for identifying "valid fitted values"

The following section provides a description of the 4-step procedure devised to identify whether the person-specific model fitted values provided a valid description of the habit trajectory.

1. **Identify cases where fitted values decrease over time based on model parameters.** In the case of best-fitting polynomial models, this distinction was made on the linear parameter. While the linear parameter alone does not definitively describe the overall trend of polynomial models, within the present sample a positive linear parameter distinguished whether an overall decrease in habit strength was absent.
2. **Identify cases where model selection is not impacted by individual observations.** To test the robustness of model selection, models were rerun with shortened timeseries for cases with long gaps of missing values (≥ 15 days) in the second half of the time series. Here, singular observations that followed a long missing gap were removed to test whether these single observations influenced model selection. In cases where the best fitting model changed, model fitted values were considered nonvalid.
3. **Identify cases where the best fitting model shows sufficient absolute fit.** Based on visual inspection, the root-mean-square-error (RMSE) cut-off value of 0.33 was deemed to distinguish accurate from inaccurate models well. Substantially, as RMSE is on the same scale as the dependent variable (Self-report Behavioral Automaticity Index; SRBAI), this cut-off indicates that the average difference between the observed and predicted value is approximately one third of a Likert point (on a 5-point Likert scale).
4. **Identify cases where time series do not contain missing gaps of observations longer than 21 days in length.** Visual inspection suggested long missing gaps to often be accompanied by

seemingly unrealistic fitted values of habit strength. Consequently, models of time series containing missing gaps of at least three weeks (21 days) in length were considered nonvalid.

1.5 Group-level models

Multilevel modeling was conducted using the R package lme4 (Bates et al., 2015).

1.6 Divergence from protocol

First, the best fitting person-specific model was determined with the BIC index, opposed to RMSE. This deviation was done to favor parsimony in model selection. Second, the logistic model used is a 4-parameter log-logistic model, opposed to the 5-parameter Richard's curve (Richards, 1959) logistic model. This deviation was due to a lack of success in fitting the Richard's curve. Lastly, explorative methods were used to address the research question of comparing the habit decay process between behavioral groups.

2. Results

2.1 Sample characteristics

See Figure S2 for flow chart of participant retention. Comparison of sociodemographic characteristics between participants included for analysis and those excluded indicated no group difference based on age, gender, Body Mass Index (BMI), civil status, occupational status, or highest level of education. Regarding the day 7 survey, intention to change behavior was higher in the sample included for analysis (*Mdn* = 3.5. *SD* = 0.76; *n* = 194) compared to the excluded sample (*Mdn* = 3.0, *SD* = 0.92; *n* = 58). Also, among participants who created an implementation intention on day 7, there was a difference in the strategy chosen by participants in the included and excluded samples. Specifically, a higher proportion of participants in the excluded sample selected the discontinuity strategy (19%, *n* =11) compared to the analyzed sample (7%, *n* =13), and a lower proportion of the excluded sample (26%, *n* = 15) selected the inhibition strategy compared to the analyzed sample (35%, *n* = 68). For comprehensive baseline characteristics of the sample included

for analysis see Table S1; here, concerning group comparisons, post-hoc pairwise behavioral group differences for highest level of education and occupational status could not be confirmed after Bonferroni's adjustment of p-values.

Figure S2. Flow diagram of participant retention. Note. SRBAI*: Self-Report Behavioral Automaticity Index

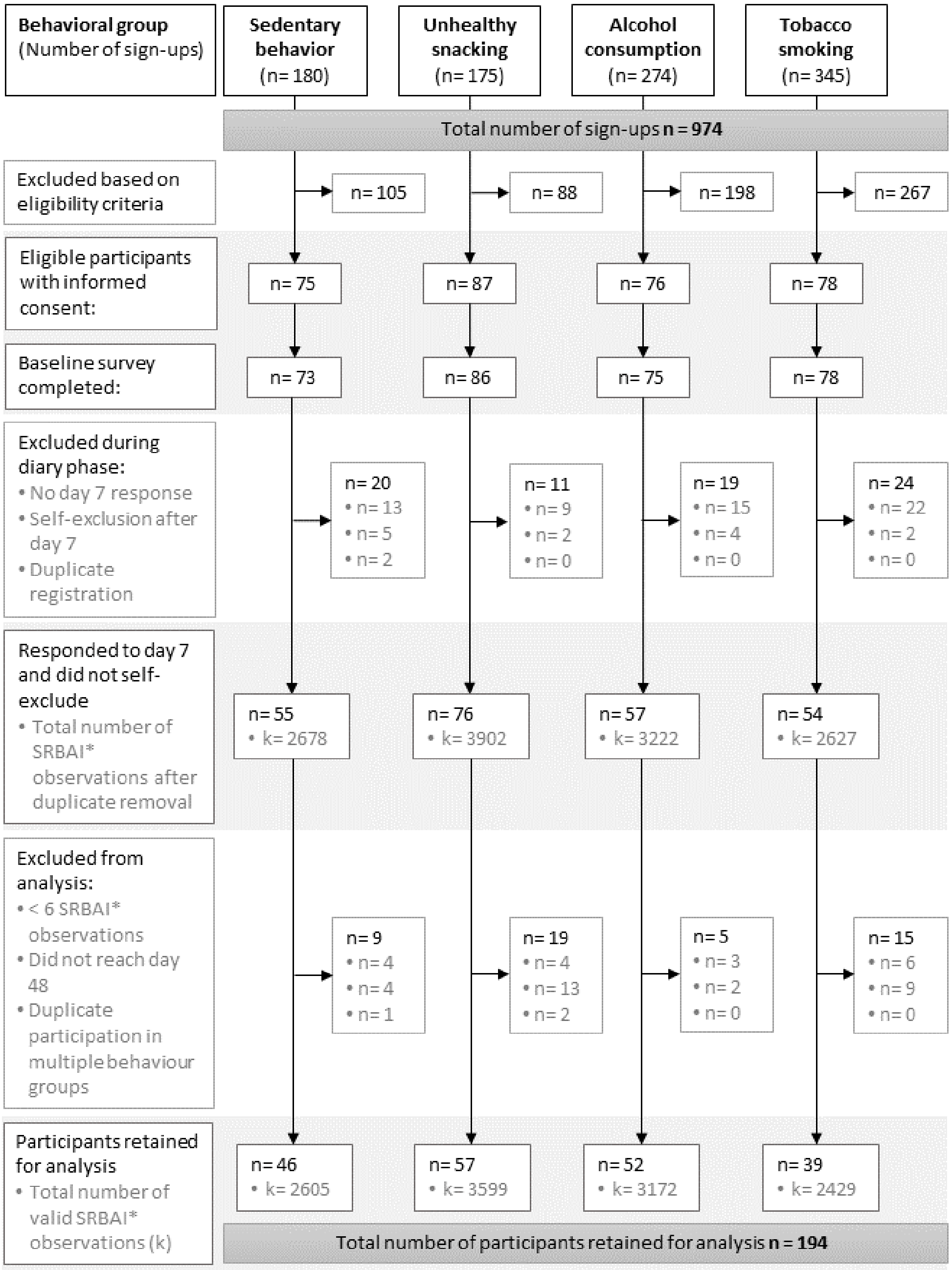

Table S1. Comprehensive baseline sample characteristics by behavioral group (*N* = 194).

	N	Overall	SB, *N* = 46	US, *N* = 57	AC, *N* = 52	TS, *N* = 39	*p*
**Baseline variables**							
Age	194	39 (32, 49)	34 (30, 42)	38 (30, 45)	44 (36, 53)	40 (34, 51)	<0.001
Gender	183						<0.001
Female		137 (75%)	37 (84%)	49 (89%)	24 (51%)	27 (73%)	
Male		45 (25%)	6 (14%)	6 (11%)	23 (49%)	10 (27%)	
Other		1 (0.5%)	1 (2.3%)	0 (0%)	0 (0%)	0 (0%)	
BMI	183	23.5 (21.8, 27.5)	22.1 (20.8, 25.3)	24.8 (21.9, 27.9)	24.7 (22.0, 27.9)	23.5 (22.0, 26.6)	0.109
Civil status	183						0.133
In relationship		66 (36%)	22 (50%)	22 (40%)	13 (28%)	9 (24%)	
Married		55 (30%)	10 (23%)	20 (36%)	16 (34%)	9 (24%)	
Single		39 (21%)	8 (18%)	9 (16%)	11 (23%)	11 (30%)	
Divorced		21 (11%)	3 (6.8%)	4 (7.3%)	6 (13%)	8 (22%)	
Reg. relationship		1 (0.5%)	1 (2.3%)	0 (0%)	0 (0%)	0 (0%)	
Widowed		1 (0.5%)	0 (0%)	0 (0%)	1 (2.1%)	0 (0%)	
Highest level of edu.	183						0.003
University studies		105 (57%)	32 (73%)	30 (55%)	27 (57%)	16 (43%)	
Vocational training		55 (30%)	7 (16%)	14 (25%)	19 (40%)	15 (41%)	
High school / voc. dipl.		17 (9.3%)	4 (9.1%)	10 (18%)	1 (2.1%)	2 (5.4%)	
Secondary school dipl.		6 (3.3%)	1 (2.3%)	1 (1.8%)	0 (0%)	4 (11%)	
Primary school dipl.		0 (0%)	0 (0%)	0 (0%)	0 (0%)	0 (0%)	
Occupation	183						0.043
Employed		145 (79%)	34 (77%)	44 (80%)	42 (89%)	25 (68%)	
Homemaker		13 (7.1%)	2 (4.5%)	6 (11%)	0 (0%)	5 (14%)	
Student		11 (6.0%)	6 (14%)	3 (5.5%)	1 (2.1%)	1 (2.7%)	
Retired		10 (5.5%)	1 (2.3%)	2 (3.6%)	3 (6.4%)	4 (11%)	
Unemployed		3 (1.6%)	1 (2.3%)	0 (0%)	1 (2.1%)	1 (2.7%)	
(Re)training		1 (0.5%)	0 (0%)	0 (0%)	0 (0%)	1 (2.7%)	

Note. Continuous variables reported with Median (interquartile range) and p-values based on Kruskal-Wallis rank sum test; Categorical variables reported with n(%) and p-values based on Fisher's Exact Test for Count Data with simulated p-value (based on 2000 replicates); SB: Sedentary behavior; US: Unhealthy snacking; AC: Alcohol consumption; TS: Tobacco smoking; BMI: Body Mass Index; SRBAI: Self-Report Behavioral Automaticity Index; reg.: registered; edu.: education; voc.: vocational; dipl.: diploma

Examples of cues selected by participants on day 7 from the sample retained for analysis are displayed in Table S2. These are examples of cues in response to which participants wanted to change their habitual target behavior. Of note, the implementation intentions participants formulated often incorporated multiple cue categories that were not all evident in the initial cue selected (e.g. *If I come home stressed after work…*).

Table S2. Examples of cues selected by participants by behavioral group.

	Behavioral group			
Cue category	Sedentary behavior	Unhealthy snacking	Alcohol consumption	Tobacco smoking
**Physical context**<br>Cues related to the physical environment or objects	- Office chair<br>- Train	- Kitchen<br>- Shop	- Sports on TV	- In the car<br>- Alcohol
**Social context**<br>Cues relating to the person(s) present or absent	- Lunch in a group<br>- Meeting friends	- Being alone<br>- Being in the presence of someone eating snacks	- With a friend in the evening<br>- Alone at home	- With people who smoke<br>- Alone at home
**Emotion / Cognition**<br>Cues related to emotions, physical sensations or thoughts	- Tired<br>- Desire to relax	- Desire for something sweet<br>- Stress	- Stress<br>- Boredom	- Boredom<br>- Being annoyed
**Event / temporal context**<br>Cues such as actions, routines, events that are about to start, are taking place or have already taken place. Also temporal information such as times of day	- Breakfast<br>- Watching TV	- Lunch break<br>- At 13:00	- After work<br>- Dinner	- Morning coffee<br>- Waiting for the bus

Note. Definitions to cue categories are provided in the left column. Cue categories were created inductively based on participants' responses. The examples provided are not a comprehensive overview of cues selected.

2.2 Description of missing SRBAI observations

Concerning missing SRBAI observations in the sample included in analyses, one participant did not have any missing SRBAI observations. For the remaining 193 participants the mean number of missing SRBAI observations was 24 (29 %; out of maximum 84). The median longest missing gap for the time series was 3 days and ranged from 0 to 62 days. For descriptive information about missing SRBAI observations after imputation see Table S3.

Table S3. Descriptive statistics of missing SRBAI values in time series (*N* = 194) after imputation.

	Mean (*SD*)	Median (min, max)
Percentage missing	21.19 (25.56)	7.06 (0, 90.59)
Number of missing gaps	3.07 (3.06)	2 (0, 15)
Average missing gap size (days)	4.95 (7.02)	2.33 (0, 42)
Longest missing gap (days)	10.14 (14.45)	3 (0, 62)

Note. SRBAI = Self-Report Behavioral Automaticity Index; *SD* = Standard deviation; SRBAI was measured daily for 84 days. Only non-consecutive missing values were imputed.

2.3 Person-specific modelling

Table S4 displays the frequencies of best-fitting person-specific models by behavioral group. Findings show that the asymptotic and logistic were most often the best fitting models across the sample. Concerning the absolute fit (RMSE) of best fitting models, a significant difference between the models was evident (see Table S5). Post-hoc pairwise comparison revealed that RMSE of the logistic model was significantly smaller than that of the linear and quadratic models, indicating better absolute fit. Percentage of missing values and longest missing gap of SRBAI measurements did not significantly differ between the best fitting person-specific model types (see Table S6).

Table S4. Frequencies (%) of best fitting person-specific models across behavioral groups (*N*=194).

Model	Overall	SB, *N* = 46	US, *N* = 57	AC, *N* = 52	TS, *N* = 39	*p*
Constant	15 (7.7%)	5 (11%)	3 (5.3%)	4 (7.7%)	3 (7.7%)	0.556
Linear	19 (9.8%)	4 (8.7%)	9 (16%)	3 (5.8%)	3 (7.7%)	
Quadratic	24 (12%)	5 (11%)	7 (12%)	6 (12%)	6 (15%)	
Cubic	31 (16%)	7 (15%)	8 (14%)	13 (25%)	3 (7.7%)	
Asymptotic	52 (27%)	16 (35%)	15 (26%)	9 (17%)	12 (31%)	
Logistic	53 (27%)	9 (20%)	15 (26%)	17 (33%)	12 (31%)	

Note. *p*-value based on Fisher's Exact Test for Count Data; SB: Sedentary behavior; US: Unhealthy snacking; AC: Alcohol consumption; TS: Tobacco smoking.

Table S5. Distribution of RMSE across best-fitting person specific models (*N*=194).

Model	*N*	Median (*IQR*)	Range	p
Constant	15	0.30 (0.17, 0.39)	0.04 - 1.33	<0.001
Linear	19	0.38 (0.28, 0.52)	0.11 - 0.71	
Quadratic	24	0.36 (0.21, 0.55)	0.08 - 0.88	
Cubic	31	0.30 (0.20, 0.37)	0.08 - 0.57	
Asymptotic	52	0.23 (0.12, 0.41)	0.03 - 0.71	
Logistic	53	0.19 (0.11, 0.27)	0.02 - 0.96	

Note. RMSE: Root-Mean-Square Error; IQR: Interquartile range; *p*-value based on Kruskal-Wallis rank sum test.

Table S6. Median (interquartile range) of missing value statistics across best fitting person-specific models (*N* =194).

Missing value statistic	Overall	Constant, *N* = 15	Linear, *N* = 19	Quadratic, *N* = 24	Cubic, *N* = 31	Asymptotic, *N* = 52	Logistic, *N* = 53	*p*
Percentage Missing	7 (2, 35)	19 (0, 54)	21 (2, 52)	8 (2, 47)	7 (0, 26)	9 (2, 28)	5 (2, 35)	0.847
Longest missing gap (days)	3 (2, 15)	3 (0, 39)	3 (2, 19)	2 (2, 18)	2 (0, 10)	3 (2, 12)	3 (2, 7)	0.874

Note. *p*-values based on Kruskal-Wallis rank sum test.

2.3.1 Sensitivity analyses

Two types of sensitivity analysis were conducted. First, person-specific models were fitted without usage of imputed SRBAI values. The frequencies of best fitting models remained relatively unchanged, with asymptotic and logistic models being the most common best fitting models. However, the asymptotic model was more frequently the best fitting model (n = 57) compared to the logistic (n = 43). See Table S7 for further details.

Table S7. Frequencies (%) of best fitting person-specific models without missing value imputation across behavioral groups (*N*=194).

Model	Overall	SB, *N* = 46	US, *N* = 57	AC, *N* = 52	TS, *N* = 39	*p*-value
Constant	17 (8.8%)	4 (7.7%)	5 (11%)	5 (8.8%)	3 (7.7%)	0.876
Linear	23 (12%)	3 (5.8%)	6 (13%)	10 (18%)	4 (10%)	
Quadratic	25 (13%)	7 (13%)	5 (11%)	7 (12%)	6 (15%)	
Cubic	27 (14%)	11 (21%)	7 (15%)	6 (11%)	3 (7.7%)	
Asymptotic	57 (29%)	13 (25%)	14 (30%)	16 (28%)	14 (36%)	
Logistic	45 (23%)	14 (27%)	9 (20%)	13 (23%)	9 (23%)	

Note. *p*-value based on Fisher's Exact Test for Count Data; SB: Sedentary behavior; US: Unhealthy snacking; AC: Alcohol consumption; TS: Tobacco smoking.

Second, sensitivity analysis was conducted to assess the impact of updating if-then plans on day 48. On day 48 in total 59 participants (out of 194; 30 %) opted to update their if-then plan (see Table S8). To test whether updating the if-then plan on day 48 had an impact on the habit strength trajectory, linear regression models predicting habit strength by time were conducted for two time windows – the week preceding day 48 and the week following day 48. Subsequently, the change in slope was calculated based on the difference in the linear parameter of the regression models for the two time windows. Results indicated (see Table S9.) that there was not a difference in the change in slope between participants who maintained the same if-then plan and those who updated their if-then plan.

Table S8. Frequencies (%) of how if-then plan strategies were changed on day 48 (N = 59)

Day 7 plan strategy	Day 48 plan strategy		
	Substitution	Inhibition	Discontinuation
Substitution	21 (35.6%)	10 (16.9%)	7 (11.9%)
Inhibition	5 (8.5%)	12 (20.3%)	2 (3.4%)
Discontinuation	1 (1.7%)	1 (1.7%)	0 (0%)

Note. Updating the if-then plan was optional. 59 out of 194 participants changed their plan on day 48.

Table S9. Median (interquartile range) of change in linear slope of habit strength from the week proceeding day 48 to the following week by if-then plan update status (N = 163).

	Overall (N = 163)	Plan unchanged (N = 112)	Plan updated (N = 51)	p
Change in slope	0.000 (-0.029, 0.026)	0.000 (-0.027, 0.026)	0.000 (-0.036, 0.030)	0.318

Note. p-value based on Kruskal-Wallis rank-sum test. Participants with > 0 observations for both time windows (days 41-48 and 48-55) were included in analysis.

2.4. Exploration of habit strength trajectories in relation to cue-behavior performance

The SHRI and SRBAI have been successfully used to investigate within-person fluctuations in self-reported habit strength during habit formation, but not yet for habit decay. One may ask, therefore, whether it is plausible that individuals can reflect on their habit when they do not perform the behavior at the occurrence of the cue. We explored this with the following additional analyses. To establish whether and to what extent the perception of habit strength reflects the performance of the target behavior when encountering the cue, additional data visualizations were generated. This exploration is not a comprehensive assessment of the data. The purpose is to provide insights to how behavior potentially influences the perception of habit in the context of trying to degrade a habit. This serves to inform about the validity of the present measurement of habit strength.

2.4.1 Methods

For this exploration, items from the end-of-day e-diary (days 8 to 91) questionnaire addressing cue-encounters and performance of the target behavior in response to cue encounters were utilized. Cue-encounters were assessed daily with one single item that referenced the participant's self-selected cue "Have you experienced the (*cue*) situation you chose today?", with 3 answer options: 0) *no*, 1) *yes, once*, and 2) *yes, several times*. Subsequently if participants reported having encountered their cue, they were asked about performing their target behavior with one single item, for example (for the tobacco smoking group) "*Did you smoke in the situation you selected (cue)?*". The response options for this latter item depended on the participant's response to the previous cue-encounters item. Specifically, if the participant reported encountering the cue once, the response options for the behavioral performance were 0) *no* and 1) *yes*. If the participant reported encountering the cue multiple times, the response options were 0) *no*, 1) *yes, sometimes* and 2) *yes, always.* The previously described items were added to the e-diary questionnaire approximately one month after the launch of study recruitment, and consequently these items were available for the full duration of study participation for 49 participants in total.

To facilitate interpretation of the present analysis, daily cue-encounters and cue-behavior performance item responses were used to compute a 3-level categorical variable indicating whether 1) the cue had not been encountered, 2) the cue had been encountered and the behavior was subsequently not performed, and 3) the cue had been encountered and the behavior was subsequently performed at least sometimes. Subsequently, habit strength observations were plotted over time (observed values only for days 8 to 91). The 3-level categorical cue-behavior performance variable was added to the plots to visualize how this potentially covaried with habit

strength. This procedure was done with participant data where cue-encounters and cue-behavior performance could be reported for the full time series (*n* = 49).

2.4.2 Results

The below Figure S3. panel plot displays 49 person-specific habit strength time series, where observations are color coded based on whether the cue was not encountered on that day (green), the cue was encountered and the behavior not performed (blue), or the cue was encountered and the behavior was performed at least sometimes (red). The individual plots are ordered based on the number of days when the cue-behavior was performed. Here, the first row contains time series where the cue-behavior was performed on 0 to 1 days in total, whereas on the last row the cue-behavior was performed on 43 to 72 days in total.

The plots in Figure S3. indicate that the association between cue-behavior performance and habit strength is idiosyncratic. Based on visual inspection, generalizations across participants cannot be made as to how habit strength may covary with cue-behavior performance. Findings suggest habit strength is not merely a reflection of cue-behavior performance or lack thereof. Importantly, trends are not evident that would suggest a difference in habit strength when the cue-behavior is performed compared to when it is not performed.

Results show that for some participants, habit gradually decreases while the behavior was relatively consistently not performed at cue encounters (e.g. 1st row, plots 2 & 3; 2nd row plots 2 & 3; 4th row plot 3). Other participants show limited change in habit strength while the behavior was relatively consistently not performed at cue encounters (e.g. 3rd row, plot 4 & 5). Lastly, there were less visible associations between cue-behavior performance and habit strength for some participants (e.g. 1st row, plot 7; 7th row, plot 6).

Figure S3. Panel plot of person-specific habit strength trajectories and cue-behavior performance ordered by performance frequency (N = 49).

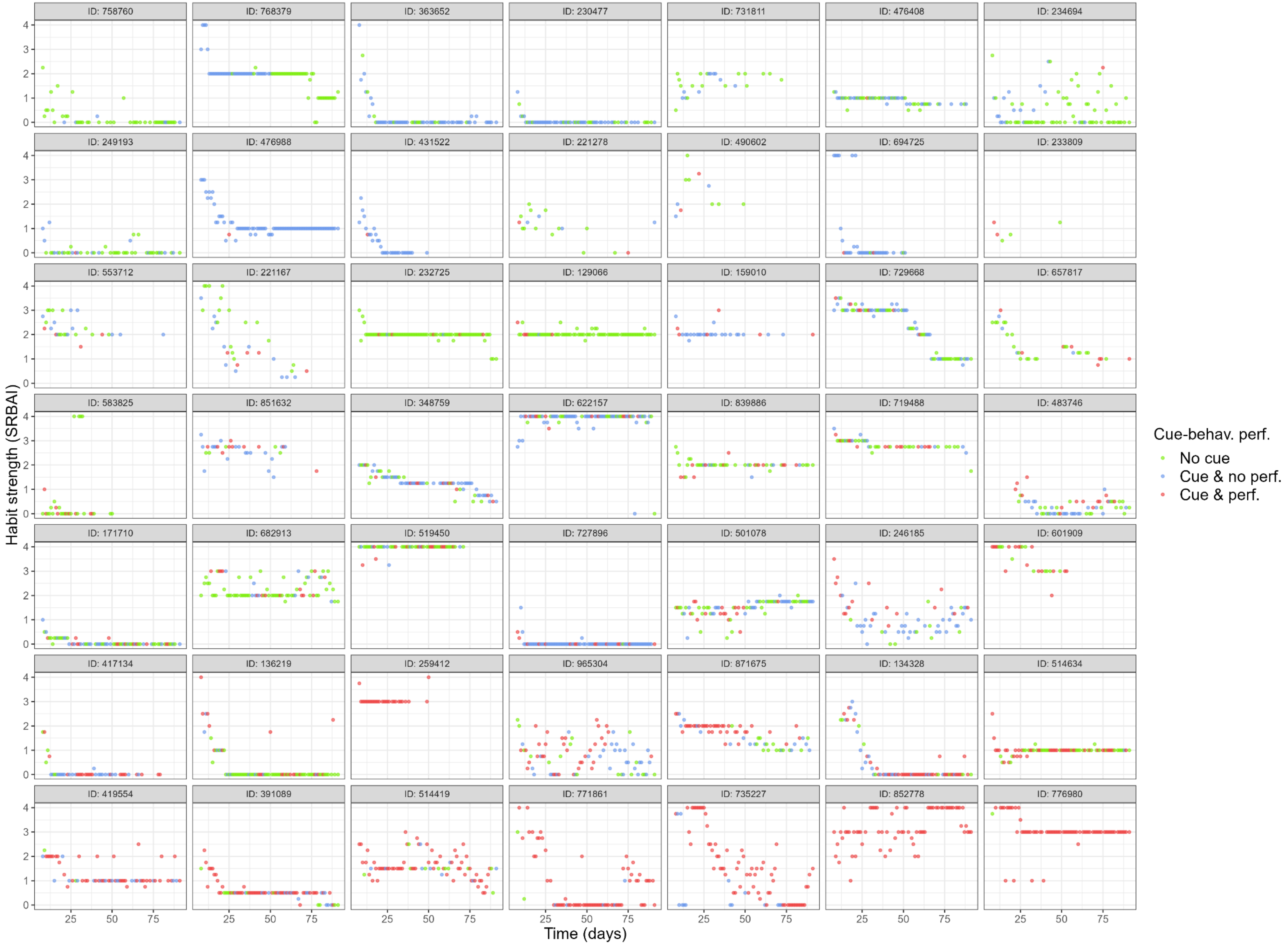

Note. Cue-behavior performance does not account for multiple cue encounters on the same day. SRBAI = Self-Report Behavioral Automaticity Index; Cue-behav. perf = Cue-behavior performance

2.4.3 Discussion

Overall, the present explorative data visualization of habit strength and cue-behavior performance provides initial support for the use of self-report habit strength in the context of habit degradation. The rationale for this conclusion lies in one key observation. Namely, findings show that habit strength (i.e. perceived automaticity) is distinct from cue-behavior performance. Within the presented plots there are no instances where habit strength would merely reflect the cue-behavior performance of that day. Without going into substantial interpretation of the plots, this indicates that it is feasible for an individual to distinguish between (non-)performance of habitual behavior, and the strength of automaticity of the underlying habit. This underscores a key facet to validity, as contemporary definitions of habit denote the cognitive representation of the cue-behavior association to be the key component of a habit, where habitual behavior is a potential outcome of an underlying habit (Fleetwood, 2021; Gardner, 2015).

Establishing that habit strength and cue-behavior performance are distinct within the context of degrading a habit is an essential first step for determining measurement validity of self-report habit strength. More research is needed to deepen our understanding of potential boundary conditions for when self-report habit strength can accurately be assessed while degrading a habit. For example, it is relevant to determine how irregular or infrequent cue encounters, and inconsistent responding to cue encounters (e.g. due to multiple daily cue encounters) may potentially influence the perception of automaticity. These latter cue-behavior performance characteristics may provide insight into some of the unexplained variation observed in habit strength; however, such an investigation falls outside of the scope of the present paper. Person-specific data visualization combined with think-aloud qualitative methods may be a fruitful avenue for future intensive longitudinal research on habit degradation to further explore validity.

2.5 Group-level modelling

Multilevel modelling indicated the asymptotic model with random effects estimated for all parameters to have the best parsimonious fit, followed by the cubic model with all random effects estimated. The fixed effects of both these multilevel models were similar in shape as shown in Figure S4.

Figure S4. Plot of two-level asymptotic and cubic model fixed effects.
Note: SRBAI = Self-Report Behavioral Automaticity Index. $N = 194$.

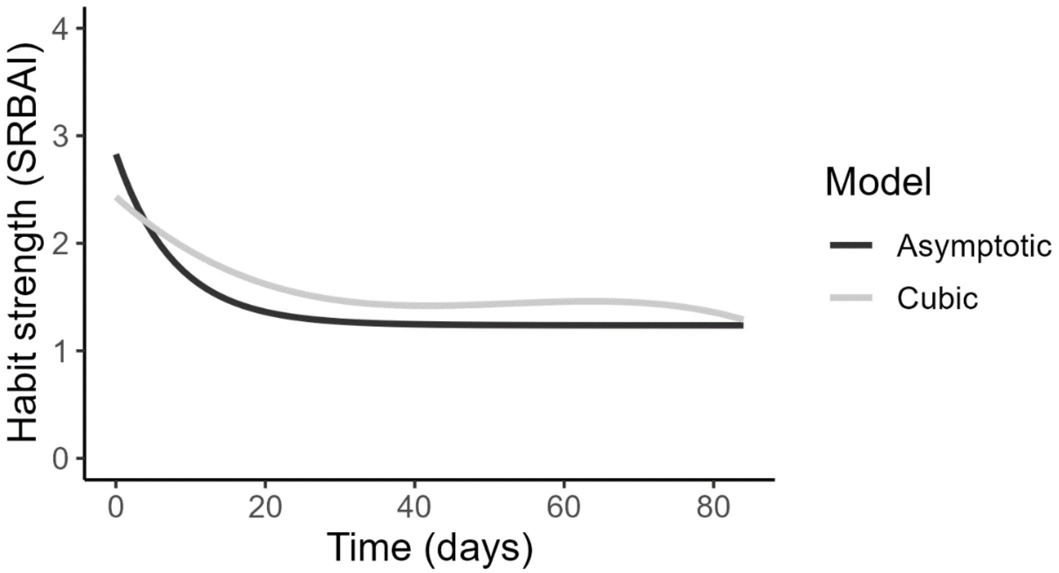

2.5.1 Behavioral group differences

Using the asymptotic model, behavioral group differences were investigated by adding behavioral group random effects. Results indicated allowing for initial habit strength to vary by behavioral group improved model fit. Results also gave indication that allowing for asymptote to vary by behavioral group improved model fit, but not for the rate of change (see Table S10). Allowing for both initial habit strength and asymptote to vary by behavioral group (not displayed) did not improve model fit by any indices.

Using the cubic model, behavioral group differences were investigated by adding intercept by behavioral group main effects and interaction terms to the model. Results indicated adding a main effect (i.e. intercept) for each behavioral group improved model fit for sedentary behavior and alcohol consumption groups (Table S10). See Table S11 for estimates of the cubic multilevel models with behavioral group main effects; here it can be seen that the intercept is significantly higher for the sedentary behavior group and lower for the alcohol consumption group compared to the average intercept of the other behaviors combined. See Table S12 for estimates of the cubic multilevel models with interaction terms for the time parameters for each behavioral group; here it can be seen that the estimates of the interaction terms for the linear and polynomial time parameters are not significant as the confidence intervals include the value 0.

Table S10. Comparison of two- and three-level models predicting habit strength with different time parameters (N = 194).

Models	*df*	*AIC*	*BIC*	Deviance	$\Delta\chi^2$	Δdf	p(Δχ2)
**Asymptotic**							
M1: Between person random effects for *R0*, *ASYM*, *LRC* (two-level)	7	14898	14950	14884			
M1 + behavioral group random effect for *R0*	8	14841	14901	14825	58.915	1	<.001
M1 + behavioral group random effect for *ASYM*	8	14896	14956	14880	3.9729	1	0.046
M1 + behavioral group random effect for *LRC*	8	14900	14960	14884	0.0034	1	0.9532
**Cubic**							
M2: Between person random effects for β0, β1, β2, β3 (two-level)	15	15129	15241	15099			
M2 + Group main effect (sedentary behavior)	16	15102	15222	15070	28.878	1	<.001
M2 + Group main effect (unhealthy snacking)	16	15128	15247	15096	3.3811	1	0.066
M2 + Group main effect (alcohol consumption)	16	15107	15226	15075	24.327	1	<.001
M2 + Group main effect (tobacco smoking)	16	15127	15247	15095	3.6235	1	0.057

Note. Asymptotic model parameters: *R0* = response on day 0, *ASYM* = asymptote, *LRC* = natural logarithm of rate constant; Cubic model parameters: β0 = Intercept, β1 = Linear time, β2 = Quadratic time, β3 = Cubic time; *BIC* = Bayesian Information Criterion; *AIC* = Akaike Information Criterion

Table S11. Multilevel cubic models with behavioral group main effects ($N = 194$).

	Fixed effects				RE
Parameters	Estimate	*SE*	*t*-value	95% *CI*	*SD*
Intercept (β0)	2.24	0.08	29.72	[2.09, 2.38]	0.91
Time (β1)	-3.03	0.26	-11.65	[-3.55, -2.52]	3.49
$Time^2$ (β2)	2.94	0.32	9.30	[2.32, 3.57]	4.17
$Time^3$ (β3)	-0.91	0.11	-8.22	[-1.13, -0.69]	1.43
Group (Sedentary behavior)	0.82	0.15	5.63	[0.53, 1.11]	
Residual					0.39
Intercept (β0)	2.51	0.08	30.09	[2.35, 2.67]	0.98
Time (β1)	-3.03	0.26	-11.65	[-3.55, -2.52]	3.49
$Time^2$ (β2)	2.94	0.32	9.29	[2.32, 3.57]	4.17
$Time^3$ (β3)	-0.91	0.11	-8.21	[-1.13, -0.69]	1.43
Group (Unhealthy snacking)	-0.27	0.15	-1.86	[-0.56, 0.02]	
Residual					0.39
Intercept (β0)	2.62	0.08	33.75	[2.47, 2.78]	0.93
Time (β1)	-3.03	0.26	-11.65	[-3.55, -2.52]	3.49
$Time^2$ (β2)	2.94	0.32	9.29	[2.32, 3.57]	4.17
$Time^3$ (β3)	-0.91	0.11	-8.21	[-1.13, -0.69]	1.43
Group (Alcohol consumption)	-0.72	0.14	-5.12	[-1.00, -0.45]	
Residual					0.39
Intercept (β0)	2.37	0.08	29.94	[2.21, 2.52]	0.98
Time (β1)	-3.03	0.26	-11.65	[-3.55, -2.52]	3.49
$Time^2$ (β2)	2.94	0.32	9.29	[2.32, 3.57]	4.17
$Time^3$ (β3)	-0.91	0.11	-8.21	[-1.13, -0.69]	1.43
Group (Tobacco smoking)	0.32	0.17	1.93	[-0.01, 0.64]	
Residual					0.39

Note. Time is scaled to vary from 0 to 1.7.; Group interaction term is coded 0 or 1, where 1 refers to the specified behavioral group and 0 all other behavioral groups combined. RE: random effects; *CI*: confidence interval; Confidence intervals computed using Wald t-distribution with Satterthwaite approximation; *SD*: standard deviation.

Table S12. Fixed effect estimates [95% confidence interval] for multilevel cubic models with interaction terms for each behavioral group.

	Sedentary behavior			Unhealthy snacking		
Parameters	M2a	M2b	M2c	M2a	M2b	M2c
Intercept	2.23 [2.08, 2.38]	2.22 [2.07, 2.37]	2.21 [2.06, 2.36]	2.52 [2.36, 2.69]	2.52 [2.35, 2.69]	2.53 [2.36, 2.69]
Time	-3.01 [-3.52, -2.49]	-2.96 [-3.48, -2.43]	-2.84 [-3.42, -2.25]	-3.06 [-3.58, -2.55]	-3.06 [-3.59, -2.52]	-3.20 [-3.81, -2.59]
$Time^2$	2.95 [2.32, 3.57]	2.92 [2.29, 3.55]	2.76 [2.05, 3.47]	2.94 [2.32, 3.57]	2.94 [2.31, 3.57]	3.14 [2.39, 3.88]
$Time^3$	-0.91 [-1.13, -0.69]	-0.91 [-1.13, -0.69]	-0.86 [-1.10, -0.61]	-0.91 [-1.13. -0.69]	-0.91 [-1.13, -0.69]	-0.98 [-1.24, -0.72]
Group	0.86 [0.57, 1.15]	0.90 [0.59, 1.21]	0.92 [0.61, 1.23]	-0.31 [-0.60, -0.01]	-0.30, [-0.61, 0.01]	-0.32 [-0.63, -0.01]
Group by time	-0.12 [-0.28, 0.04]	-0.32 [-0.82, 0.18]	-0.83 [-2.04, 0.37]	0.09 [-0.05, 0.24]	0.07 [-0.39, 0.54]	0.57 [-0.55, 1.70]
Group by $time^2$		0.10 [-0.14, 0.35]	0.79 [-0.68, 2.26]		0.01 [-0.21, 0.23]	-0.65 [-2.02, 0.72]
Group by $time^3$			-0.24 [-0.76, 0.27]			0.23 [-0.24, 0.71]
	Alcohol consumption			Tobacco smoking		
Parameters	M2a	M2b	M2c	M2a	M2b	M2c
Intercept	2.63 [2.48, 2.79]	2.64 [2.48, 2.79]	2.64 [2.49, 2.80]	2.36 [2.20, 2.52]	2.37 [2.21, 2.52]	2.36 [2.20, 2.52]
Time	-3.05 [-3.57, -2.54]	-3.08 [-3.61, -2.56]	-3.21 [-3.81, -2.61]	-3.01 [-3.53, -2.50]	-3.04 [-3.57, -2.52]	-2.91 [-3.48, -2.34]
$Time^2$	2.94 [2.32, 3.57]	2.96 [2.33, 3.58]	3.12 [2.39, 3.85]	2.94 [2.32, 3.57]	2.96 [2.33, 3.58]	2.78 [2.08, 3.48]
$Time^3$	-0.91 [-1.13, -0.69]	-0.91 [-1.13, -0.69]	-0.97 [-1.22, -0.71]	-0.91 [-1.13, -0.69]	-0.91 [-1.13, -0.69]	-0.85 [-1.09, -0.60]
Group	-0.75 [-1.04, -0.47]	-0.78 [-1.08, -0.48]	-0.79 [-1.10, -0.49]	0.36 [0.03, 0.69]	0.32 [-0.03, 0.67]	0.35 [0.00, 0.70]
Group by time	0.08 [-0.07, 0.23]	0.19 [-0.29, 0.67]	0.66 [-0.50, 1.81]	-0.10 [-0.27, 0.06]	0.06 [-0.48, 0.59]	-0.61 [-1.89, 0.67]
Group by $time^2$		-0.06 [-0.29, 0.17]	-0.67 [-2.08, 0.74]		-0.08 [-0.34, 0.18]	0.81 [-0.75, 2.37]
Group by $time^3$			0.22 [-0.27, 0.71]			-0.31 [-0.86, 0.23]

Note: N = 194; Dependent variable is Self-Report Behavioral Automaticity Index (score range 0-4); Time rescaled to 0 - 1.72 (i.e. 1 day increase equates to ca. 0.02 increase in rescaled time); Group term coded 0, 1, where 1 refers to the specified behavioral group and 0 all other behavioral groups combined. All models have between person random effects estimated for all parameters (not displayed); Confidence intervals calculated with Satterthwaite approximation; M2a: model with interaction for linear time by group; M2b: model with interaction for quadratic time by group; M2c: model with interaction for cubic time by group.

2.5.2 Sensitivity analyses

Sensitivity analysis was conducted by rerunning the cubic multilevel models with behavioral group main effects without using missing value imputation, which confirmed previous results (see Table S13). Additionally, day 7 intention was added as a covariate to the cubic multilevel model (with imputed SRBAI values), which indicated intention to have a negligible effect despite displaying a significant interaction with the cubic time parameter (see Table S14 and Figure S5). The absence of an effect of day 7 intention on the habit degradation process is reasonable considering that intention strength varies over time (Conner & Norman, 2022).

Table S13. Multilevel cubic model predicting habit strength by time with main effect for behavior group without missing value imputation (N = 194).

	Sedentary behavior				Unhealthy snacking			
Parameters	Estimate	*SE*	95% *CI*	*p*	Estimate	*SE*	95% *CI*	*p*
Intercept	2.24	0.08	[2.10, 2.39]	<0.001	2.52	0.08	[2.35, 2.68]	<0.001
Time	-3.06	0.26	[-3.57, -2.55]	<0.001	-3.06	0.26	[-3.57, -2.55]	<0.001
$Time^2$	2.98	0.32	[2.36, 3.60]	<0.001	2.97	0.32	[2.36, 3.59]	<0.001
$Time^3$	-0.92	0.11	[-1.14, -0.71]	<0.001	-0.92	0.11	[-1.14, -0.71]	<0.001
Group	0.82	0.15	[0.53, 1.10]	<0.001	-0.27	0.14	[-0.55, 0.01]	0.061
	Alcohol consumption				Tobacco smoking			
Parameters	Estimate	*SE*	95% *CI*	*p*	Estimate	*SE*	95% *CI*	*p*
Intercept	2.63	0.08	[2.48, 2.78]	<0.001	2.37	0.08	[2.22, 2.52]	<0.001
Time	-3.06	0.26	[-3.57, -2.55]	<0.001	-3.06	0.26	[-3.57, -2.55]	<0.001
$Time^2$	2.97	0.32	[2.35, 3.59]	<0.001	2.97	0.32	[2.35, 3.59]	<0.001
$Time^3$	-0.92	0.11	[-1.14, -0.71]	<0.001	-0.92	0.11	[-1.14, -0.71]	<0.001
Group	-0.72	0.14	[-1.00, -0.45]	<0.001	0.33	0.16	[0.00, 0.65]	0.047

Note. CI: Confidence interval; Confidence intervals calculated with Satterthwaite approximation

Table S14. Multilevel cubic model predicting habit strength by time with main effect and interaction terms for day 7 intention (N = 194).

Parameters	Estimate	*SE*	*95% CI*	*p*
Intercept	2.43	0.07	[2.29, 2.57]	<.001
Time	-3.03	0.26	[-3.54, -2.52]	<.001
Time2	2.94	0.31	[2.33, 3.56]	<.001
Time3	-0.91	0.11	[-1.13, -0.70]	<.001
Intention	-0.02	0.10	[-0.21, 0.16]	0.797
Intention by time	-0.45	0.34	[-1.12, 0.23]	0.191
Intention by time2	0.82	0.41	[0.00, 1.63]	0.049
Intention by time3	-0.32	0.14	[-0.61, -0.04]	0.024

Note. Intention is grand mean centered; CI: Confidence interval; Confidence intervals calculated with Satterthwaite approximation

Figure S5. Multilevel cubic model plots with day 7 intention as covariate (N = 194).

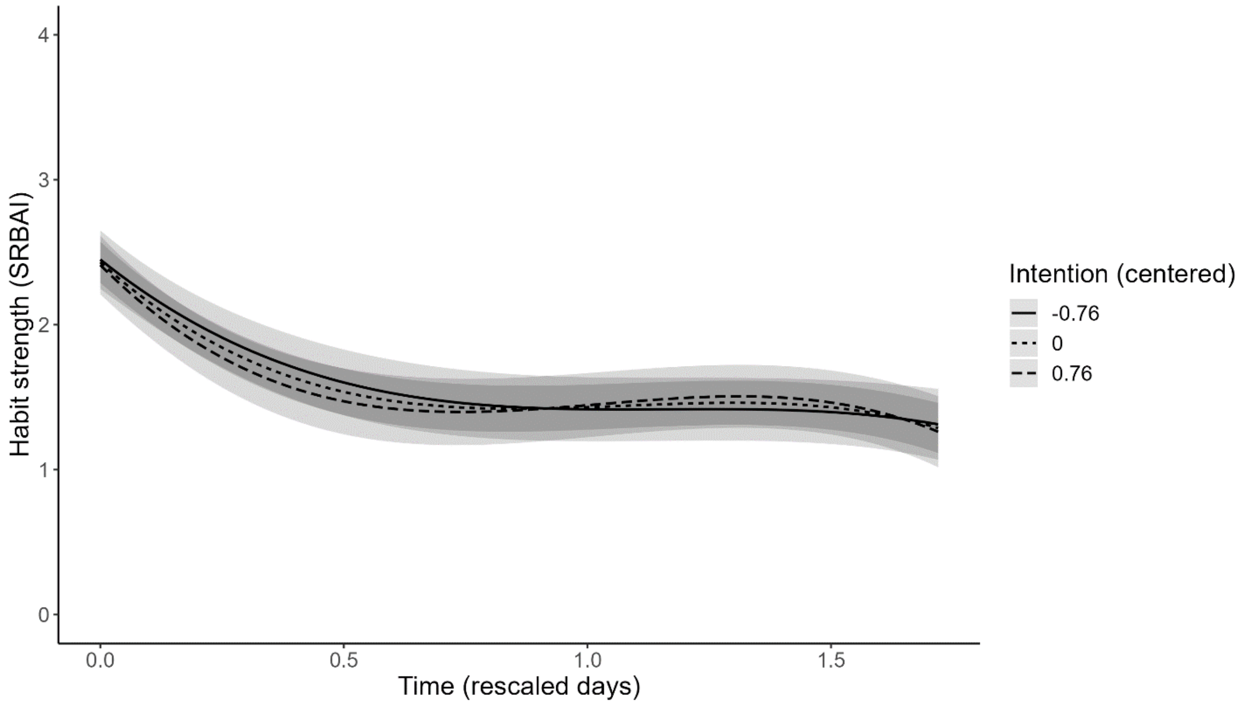

Note: Intention is grand mean centered and displayed at the mean level (0) and 1 standard deviation below (-0.76) and above (0.76) the mean. Time (days 0 to 84) has been rescaled to vary from 0 to 1.72.

References

Bates, D., Mächler, M., Bolker, B., & Walker, S. (2015). Fitting Linear Mixed-Effects Models Using **lme4**. *Journal of Statistical Software*, *67*(1). https://doi.org/10.18637/jss.v067.i01

Conner, M., & Norman, P. (2022). Understanding the intention-behavior gap: The role of intention strength. *Frontiers in Psychology*, *13*, 923464. https://doi.org/10.3389/fpsyg.2022.923464

Evans, R., Norman, P., & Webb, T. L. (2017). Using Temporal Self-Regulation Theory to understand healthy and unhealthy eating intentions and behaviour. *Appetite*, *116*, 357–364. https://doi.org/10.1016/j.appet.2017.05.022

Fleetwood, S. (2021). A definition of habit for socio-economics. *Review of Social Economy*, *79*(2), 131–165. https://doi.org/10.1080/00346764.2019.1630668

FoodDrinkEurope. (2023). *Portion guidance: The basics*. https://www.fooddrinkeurope.eu/resource/fooddrinkeurope-guidelines-portion-guidance-the-basics/

Gardner, B. (2015). A review and analysis of the use of 'habit' in understanding, predicting and influencing health-related behaviour. *Health Psychology Review*, *9*(3), 277–295. https://doi.org/10.1080/17437199.2013.876238

Inauen, J., Shrout, P. E., Bolger, N., Stadler, G., & Scholz, U. (2016). Mind the Gap? An Intensive Longitudinal Study of Between-Person and Within-Person Intention-Behavior Relations. *Annals of Behavioral Medicine*, *50*(4), 516–522. https://doi.org/10.1007/s12160-016-9776-x

Kelly, B., Smith, B., King, L., Flood, V., & Bauman, A. (2007). Television food advertising to children: The extent and nature of exposure. *Public Health Nutrition*, *10*(11), 1234–1240. https://doi.org/10.1017/S1368980007687126

Moritz, S., & Bartz-Beielstein, T. (2017). imputeTS: Time Series Missing Value Imputation in R. *The R Journal*, *9*(1), 207. https://doi.org/10.32614/RJ-2017-009

Onofri, A. (2019, January 8). Some useful equations for nonlinear regression in R. *The Broken Bridge between Biologists and Statisticians*. https://www.statforbiology.com/nonlinearregression/usefulequations#logistic_curve

Padfield, D., & Granville, M. (2020). *nls.multstart: Robust Non-Linear Regression using AIC Scores.* (Version R package version 1.2.0.) [Computer software]. https://CRAN.R-project.org/package=nls.multstart

Richards, F. J. (1959). A Flexible Growth Function for Empirical Use. *Journal of Experimental Botany*, *10*(2), 290–301. https://doi.org/10.1093/jxb/10.2.290

Verhoeven, A. A. C., Adriaanse, M. A., De Vet, E., Fennis, B. M., & De Ridder, D. T. D. (2014). Identifying the 'if' for 'if-then' plans: Combining implementation intentions with cue-monitoring targeting unhealthy snacking behaviour. *Psychology & Health*, *29*(12), 1476–1492. https://doi.org/10.1080/08870446.2014.950658

World Health Organization. (2001). *AUDIT: the Alcohol Use Disorders Identification Test. Guidelines for Use in Primary Care* (WHO/MSD/MSB/01.6a). Department of Mental Health and Substance Dependence, World Health Organization, Geneva, Switzerland. https://www.who.int/publications/i/item/WHO-MSD-MSB-01.6a
